# Cryo-EM structure of SETD2/Set2 methyltransferase bound to a nucleosome containing oncohistone mutations

**DOI:** 10.1038/s41421-021-00261-6

**Published:** 2021-05-11

**Authors:** Yingying Liu, Yanjun Zhang, Han Xue, Mi Cao, Guohui Bai, Zongkai Mu, Yanli Yao, Shuyang Sun, Dong Fang, Jing Huang

**Affiliations:** 1 Ninth People’s Hospital, Shanghai Jiao Tong University School of Medicine, Shanghai, 200125 China; 2 Shanghai Institute of Precision Medicine, Shanghai, 200125 China; 3 State Key Laboratory of Oncogenes and Related Genes, Shanghai Jiao Tong University School of Medicine, Shanghai, 200125 China; 4 Life Sciences Institute, Zhejiang University, Hangzhou, Zhejiang 310058 China; 5 State Key Laboratory of Molecular Biology, CAS Center for Excellence in Molecular Cell Science, Shanghai Institute of Biochemistry and Cell Biology, University of Chinese Academy of Sciences, Chinese Academy of Sciences, Shanghai, 201210 China; 6 Department of Oral and Maxillofacial-Head & Neck Oncology, Ninth People’s Hospital, Shanghai Jiao Tong University School of Medicine, Shanghai, 200011 China; 7 National Clinical Research Center for Oral Diseases, Shanghai, 200011 China; 8 Shanghai Key Laboratory of Stomatology & Shanghai Research Institute of Stomatology, Shanghai, 200011 China; 9 School of Life Sciences and Biotechnology, Shanghai Jiao Tong University, Shanghai, 200240 China

**Keywords:** Cryoelectron microscopy, Epigenetics

## Abstract

Substitution of lysine 36 with methionine in histone H3.3 (H3.3K36M) is an oncogenic mutation that inhibits SETD2-mediated histone H3K36 tri-methylation in tumors. To investigate how the oncohistone mutation affects the function of SETD2 at the nucleosome level, we determined the cryo-EM structure of human SETD2 associated with an H3.3K36M nucleosome and cofactor *S*-adenosylmethionine (SAM), and revealed that SETD2 is attached to the N-terminal region of histone H3 and the nucleosome DNA at superhelix location 1, accompanied with the partial unwrapping of nucleosome DNA to expose the SETD2-binding site. These structural features were also observed in the previous cryo-EM structure of the fungal Set2–nucleosome complex. By contrast with the stable association of SETD2 with the H3.3K36M nucleosome, the EM densities of SETD2 could not be observed on the wild-type nucleosome surface, suggesting that the association of SETD2 with wild-type nucleosome might be transient. The linker histone H1, which stabilizes the wrapping of nucleosome DNA at the entry/exit sites, exhibits an inhibitory effect on the activities of SETD2 and displays inversely correlated genome distributions with that of the H3K36me3 marks. Cryo-EM analysis of yeast H3K36 methyltransferase Set2 complexed with nucleosomes further revealed evolutionarily conserved structural features for nucleosome recognition in eukaryotes, and provides insights into the mechanism of activity regulation. These findings have advanced our understanding of the structural basis for the tumorigenesis mechanism of the H3.3K36M mutation and highlight the effect of nucleosome conformation on the regulation of histone modification.

## Introduction

Histone modifications play pivotal roles in a multitude of cellular processes, including transcription, DNA replication, and DNA damage repair^1^. Tri-methylation of histone H3 on lysine 36 (H3K36me3), primarily deposited by histone methyltransferase (HMT) SETD2 in mammalian cells, occurs at gene bodies of active chromatin and serves as one of the essential histone marks associated with active transcription^2,3^. SETD2 directly associates with the hyperphosphorylated C-terminal domain (CTD) repeats of RNA polymerase II (pol II) through its Set2–Rpb1 interaction (SRI) domain to deposit the H3K36me3 marks co-transcriptionally^4–7^, and participates in the physiological regulation of chromatin condensation, histone exchange, pre-mRNA splicing, DNA damage repair, etc.^8–16^.

Dysfunction of the SETD2–H3K36me3 axis has been linked to a wide range of human malignancies. Frequent loss or mutations of the *SETD2* gene have been observed in clear cell renal cell carcinoma, high-grade gliomas, esophageal squamous cell carcinoma, colorectal cancer, and acute leukemia^17–21^. Moreover, the substitution of lysine 36 with methionine in the histone H3 variant H3.3 (abbreviated as H3.3K36M) has been identified in chondroblastoma and head and neck squamous cell carcinomas^22,23^. This mutation results in loss of H3K36 methylation landscape and alteration of cancer-related gene expression^24^.

Structural and biochemical analysis of the catalytic domain of SETD2 toward an H3.3K36M peptide indicated that the oncohistone peptide occupies the substrate channel of SETD2 accompanied with the side chain of K36M inserted into the catalytic site, and inhibits the HMT activities of SETD2 against mononucleosomes^25,26^. Recent cryo-electron microscopy (cryo-EM) studies of several histone modifiers, such as human MLL and PRC2 complexes, bound to their natural nucleosome substrates revealed that the modifications of histone tails require specific recognition of nucleosome and even higher-order chromatin structures by the enzymes^27–29^. A recent cryo-EM structure of *Chaetomium thermophilum* Set2, a fungal homolog of SETD2, bound to a ubiquitinated nucleosome showed that the catalytic domain of Set2 makes extensive interactions with the N-terminus of histone H3 and the C-terminal tail of histone H2A, and stabilizes nucleosome DNA in the unwrapped conformation^30^. However, it remains poorly understood how the H3.3K36M mutation is recognized by human SETD2 at the nucleosome level and how it affects the physiological functions of human SETD2 in the context of chromatin structure.

SETD2 exclusively catalyzes H3K36 tri-methylation in mammalian cells, whereas mono- and di-methylations of H3K36 are implemented by the methyltransferases NSD1-3 and ASH1L^2,31^. By contrast, Set2, the yeast homolog of SETD2, is the sole enzyme responsible for the mono-, di-, and tri-methylations of H3K36 in yeast^32^. The functional domains of SETD2 are highly conserved in yeast Set2^33,34^. It remains unclear what determines the different activities of human SETD2 and yeast Set2.

In this work, cryo-EM analysis of human SETD2 in complex with an H3.3K36M nucleosome or a wild-type nucleosome revealed that the catalytic domain of SETD2 is stably associated with the H3.3K36M mutant nucleosome in a similar manner as observed in the fungal Set2–nucleosome structure^30^. By contrast, the EM densities of SETD2 could not be observed on the wild-type nucleosome surface, suggesting that the association of SETD2 with wild-type nucleosome might be very dynamic. The detachment of nucleosome DNA at its entry/exit site is a prerequisite for the nucleosome-binding and catalytic activities of SETD2. Accordingly, the linker histone H1, which stabilizes the wrapping of nucleosome DNA at the entry/exit sites, causes an inhibitory effect on the in vitro HMT activities of SETD2 toward nucleosomes. ChIP-Seq analysis also indicates that the genomic distribution of histone H1 is inversely correlated with that of the H3K36me3 marks. In addition, structural characterization of yeast Set2 bound to the oncohistone or wild-type nucleosome reveals an evolutionarily conserved structural framework for nucleosome-binding and also provides structural insights into the mechanism of activity regulation in eukaryotes.

## Results

### Overall structure of human SETD2 in complex with an H3.3K36M nucleosome

**Fig. 1:**
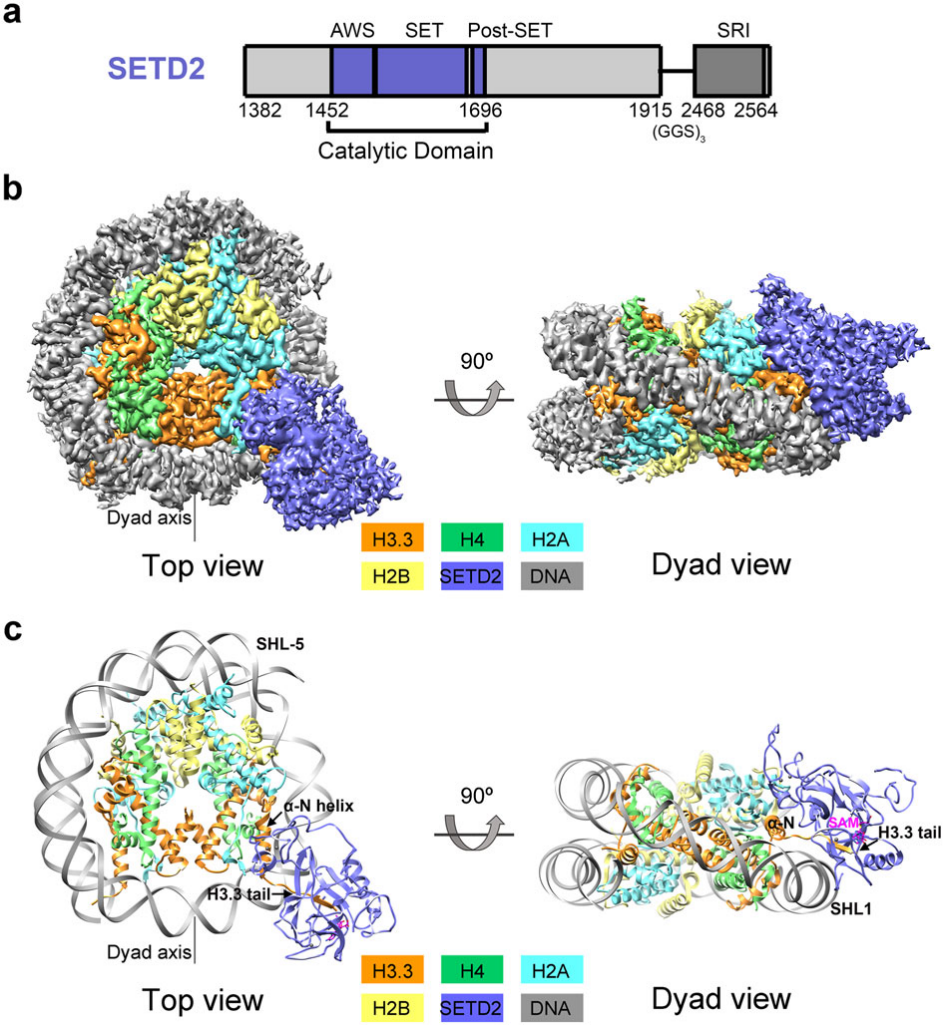
Overview of the human SETD2–hNCP^H3.3K36M^ complex structure. **a** Schematic drawing of the domain organizations of human SETD2. The color scheme is the same as that of the SETD2–hNCP^H3.3K36M^ structural model. **b** Cryo-EM density map of human SETD2–hNCP^H3.3K36M^ complex at 3.1 Å shown from two orthogonal views. The cryo-EM map was segmented and colored according to the respective components of the SETD2–hNCP^H3.3K36M^ complex. **c** Atomic model of human SETD2–hNCP^H3.3K36M^ complex shown from two orthogonal views. The catalytic domain of SETD2 binds to the α-N of histone H3.3 as well as the nucleosome DNA at SHL1.

To investigate the effects of H3.3K36M oncogenic mutation on the functions of human SETD2 at the level of the nucleosome, we reconstituted human wild-type and H3.3K36M-mutant nucleosome core particles (hereafter, hNCP^WT^ and hNCP^H3.3K36M^) in vitro and performed the cryo-EM analysis of SETD2 complexed with hNCP^WT^ and hNCP^H3.3K36M^, respectively. A truncated SETD2 construct (residues 1382 to the C-terminus, with residues 1916–2467 replaced with a (Gly–Gly–Ser)_3_ linker) that contains both the catalytic domain and the SRI domain and exhibits optimal HMT activities toward nucleosome substrates was used for the structural and biochemical analysis (Fig. 1a; Supplementary Fig. S1a–c). Electrophoretic mobility shift assays (EMSAs) indicate that SETD2 binds hNCP^WT^ or hNCP^H3.3K36M^ with comparable affinities (Supplementary Fig. S1d). Cryo-EM single-particle analysis generated a global density map of the SETD2–hNCP^H3.3K36M^ complex at a resolution of 3.1 Å (Supplementary Fig. S2 and Table S1). Masked classifications on the AWS (associated with SET) region and the active site of SETD2 further improved the local EM densities (Supplementary Fig. S2a). An atomic model of the SETD2–hNCP^H3.3K36M^ complex was built by docking available crystal structures of SETD2 and nucleosome^25^ into the EM density map, followed by manual building (Fig. 1b, c).

The SETD2–hNCP^H3.3K36M^ complex structure resembles that of the fungal Set2–nucleosome complex^30^ (Fig. 1c; Supplementary Fig. S3a). The catalytic domain of SETD2 binds to the N-terminal α helix (α-N) of histone H3.3 as well as the nucleosome DNA at superhelix location 1 (SHL1), at which the histone H3.3 tail snuggly extends through the substrate-binding channel of SETD2 (Fig. 1c). Notably, only the catalytic domain of SETD2 could be unambiguously built in the cryo-EM map of the SETD2–hNCP^H3.3K36M^ complex, which suggests that the rest of SETD2 regions might adopt flexible conformations on the mutant nucleosome (Fig. 1b, c). By contrast with the SETD2–hNCP^H3.3K36M^ complex structure, cryo-EM analysis of the SETD2–hNCP^WT^ complex indicates that the EM densities of the catalytic domain of SETD2 could not be observed on the surface of hNCP^WT^, despite that the SETD2 protein appeared to bind hNCP^WT^ similarly to hNCP^H3.3K36M^ in EMSA assays as well as in cryo-EM sample preparation (Supplementary Figs. S1d, S3b–e). This suggests that the interaction of SETD2 with hNCP^WT^ might be very dynamic, resulting in rather flexible association of the enzyme–substrate complex as seen in our structural analysis. The H3.3K36M oncohistone mutation stabilizes the association of the catalytic domain of SETD2 with the mutant nucleosome, which leads to the visualization of clear EM densities of SETD2 on the H3.3K36M nucleosome.

### Nucleosome recognition by the catalytic domain of SETD2

**Fig. 2:**
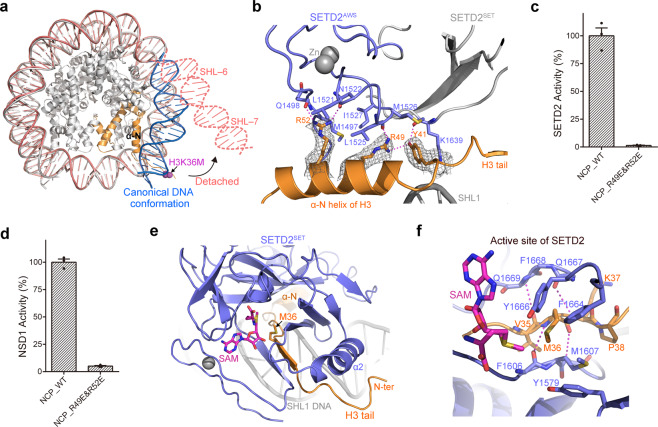
Interactions in the human SETD2–hNCP^H3.3K36M^ complex. **a** Nucleosome DNA at SHL-6 and SHL-7 is detached from the core histones in the SETD2–hNCP^H3.3K36M^ complex structure. **b** Detailed view of the recognition interface between SETD2^AWS^ and the α-N helix of histone H3.3. **c** Endpoint HMT assays carried out with wide-type or mutant hNCP, revealing that mutations of Arg49^H3.3^ and Arg52^H3.3^ to Glu drastically diminished the enzymatic activities of SETD2 towards hNCP. The HMT activities were reported for equal amounts of the SETD2 proteins. Error bars correspond to the SD of three replicate assays. Input of the HMT reactions is shown in Supplementary Fig. S4a. **d** Endpoint HMT assays carried out with wide-type or mutant hNCP, revealing that mutation of Arg49^H3.3^ and Arg52^H3.3^ to Glu severely impaired the enzymatic activities of NSD1 towards hNCP. The HMT activities were reported for equal amounts of the NSD1 proteins. Error bars correspond to the SD of three replicate assays. Input of the HMT reactions is shown in Supplementary Fig. S4c. **e** Path of the histone H3.3 tail along the SHL1 DNA, the active site and the α2 helix of SETD2. **f** Detailed view of the active site of SETD2 bound with the K36M mutation.

Similar to the structure of the fungal Set2–nucleosome complex^30^, a remarkable feature of the human SETD2–hNCP^H3.3K36M^ complex structure is that the nucleosome DNA at SHL-6 and SHL-7 is detached from the core histones to expose the binding site of SETD2 on nucleosome (Fig. 2a; Supplementary Fig. S3a). Then, the AWS domain of SETD2 (SETD2^AWS^) is attached to the α-N helix of histone H3, which confers specific interactions between SETD2 and nucleosome (Fig. 2b). At the interface, the side chains of Tyr41, Arg49 and Arg52 from histone H3 stick out and form a hydrogen-bonding network with the main chain carbonyls of Gln1498, Leu1525, Lys1639 as well as the side chain of Asn1522 from SETD2^AWS^ (Fig. 2b). The hydrophobic side chains of Met1497, Leu1521, Leu1525, Met1526 and Ile1527 from SETD2^AWS^ further constrain the side-chain conformations of Tyr41, Arg49 and Arg52 of histone H3.3 (Fig. 2b).

In vitro HMT assays of SETD2 showed that a nucleosome mutant with substitutions of Arg49^H3.3^ and Arg52^H3.3^ with glutamic acids (R49E&R52E) failed to be methylated by SETD2, which underscores the importance of these residues in SETD2-mediated H3K36 tri-methylation (Fig. 2c; Supplementary Fig. S4a). In addition, the AWS domain is commonly present N-terminally to the catalytic SET domain in multiple H3K36-specific methyltransferases, such as NSD1–3 and ASH1L (Supplementary Fig. S4b). The methyltransferase activity of NSD1 against the R49E&R52E mutant nucleosome is also dramatically diminished, suggesting that the specific recognition between AWS and the α-N helix of histone H3 might be a common mechanism involved in the methylation of the H3K36 site (Fig. 2d; Supplementary Fig. S4c).

Located N-terminally to the α-N helix of histone H3, the H3 tail region extends above the minor groove of SHL1 DNA, passes through the active site of the catalytic SET domain of SETD2, and then exits around the α2 helix of SETD2 (Fig. 2e; Supplementary Fig. S4d). The active center of SETD2–nucleosome structure resembles that observed in the crystal structures of SETD2 complexed an H3.3K36M peptide^25^ (Supplementary Fig. S4e). At the active site of SETD2, residues Val35, Met36 and Lys37 of the H3.3 tail, sandwiched within an intermolecular β-sheet structure, are stabilized through main-chain contacts with the Phe1606–Met1607–Ala1608 and Gln1667–Phe1668–Gln1669 strands of SETD2. Meanwhile, the side chain of Met36^H3.3^ is confined by the hydrophobic side chains of Tyr1579, Met1607, Phe1664 and Tyr1666 of SETD2, and its methylthio group approaches the methyl group of the cofactor *S*-adenosylmethionine (SAM) (Fig. 2f).

### Linker histone represses the deposition of H3K36me3 by SETD2

**Fig. 3:**
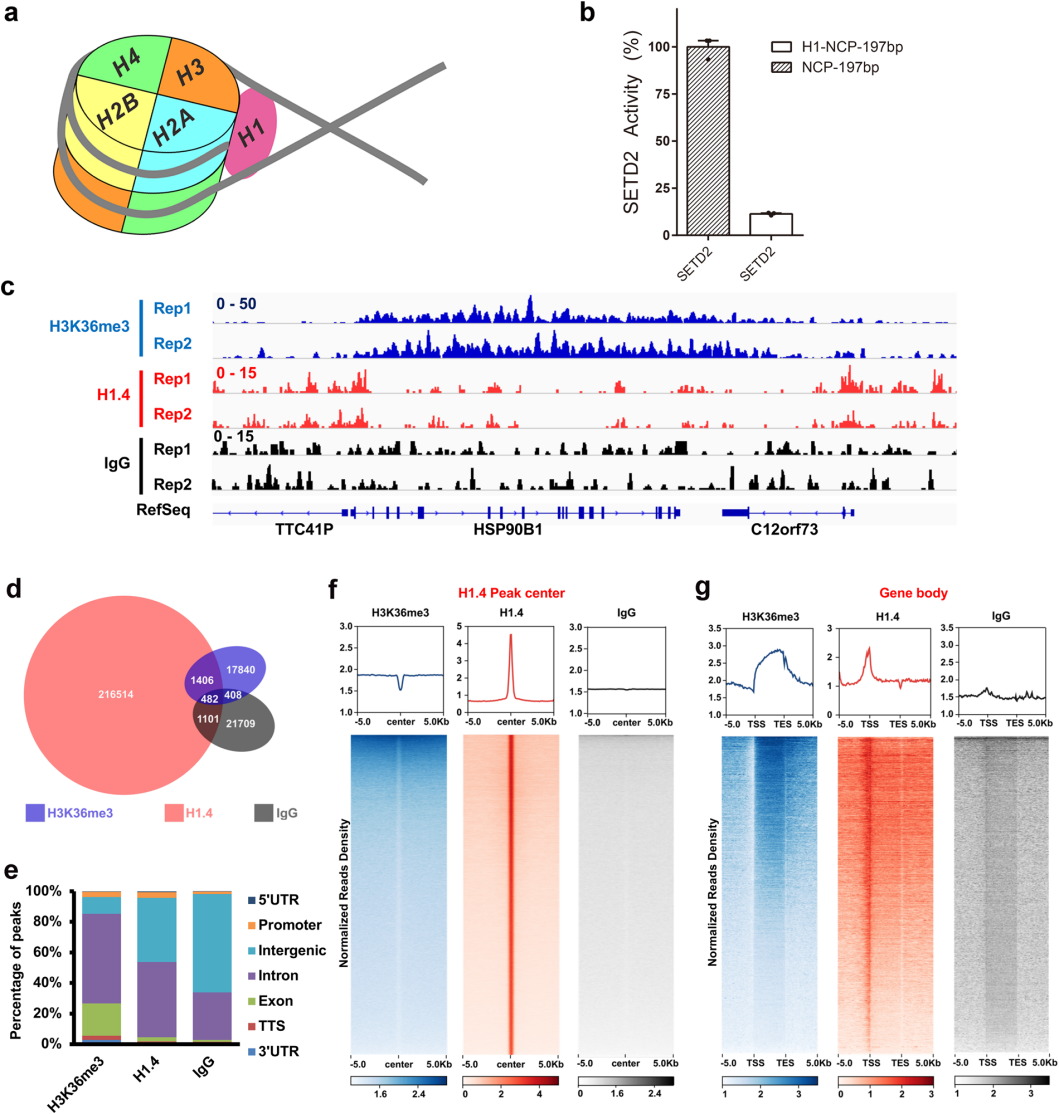
Histone H1 represses the deposition of H3K36me3 by SETD2. **a** Schematic drawing of the nucleosome bound with a linker histone H1. **b** HMT assays performed with SETD2 toward the 197-bp nucleosomes bound with histone H1.4. The input of the HMT reactions is shown in Supplementary Fig. S5a. **c** Integrative Genomics Viewer (IGV) tracks representing the distributions of H3K36me3, H1.4 and IgG ChIP-Seq results with two replicates. Rep1, replicate 1, Rep2, replicate 2. **d** Venn diagram illustration showing the overlap of H3K36me3, H1.4 and IgG peaks. The peaks in independent replicates were combined. **e** The percentages of H3K36me3, H1.4 and IgG peaks in different genomic elements. ChIP-Seq results in independent replicates were combined. The distributions were analyzed by HOMER with default parameters. **f** The enrichments of H3K36me3, H1.4 and IgG ChIP-Seq results at H1.4 peak regions. A 100-bp sliding window was used to scan 10-kb regions surrounding the peak center. Heatmaps illustrating ChIP-Seq read densities from 5 kb upstream to 5 kb downstream of the peak regions (in rows) were shown on a per-peak basis (in columns). **g** The distribution profiles of normalized H3K36me3, H1.4 and IgG read density from 5 kb upstream of the TSS to 5 kb downstream of the TES.

The attachment of SETD2 on the nucleosome requires partial unwrapping of the nucleosome DNA to expose the underlying SETD2-binding site. This particular conformation of the nucleosome often occurs during the passage of Pol II in transcription elongation^35–37^, which favors the co-transcriptional deposition of H3K36me3 by SETD2 in the gene bodies of active transcription regions. On the contrary, histone H1 interacts with the linker DNA at both the entry and exit sites of nucleosome^38–40^, which stabilizes the compact conformation of the nucleosome and might inhibit the association of SETD2 with nucleosome (Fig. 3a). To investigate the impact of linker histone on the implementation of H3K36 tri-methylation by SETD2, we assembled a 197-base pair (bp) nucleosome with the globular domain of histone H1.4 (residues 1–130) and examined the methyltransferase activities of SETD2 against the nucleosomes with or without the linker histone in vitro. As shown in Fig. 3b and Supplementary Fig. S5a, the binding of histone H1.4 resulted in an almost 90% decrease of the SETD2 activities toward the nucleosome, suggesting that the linker histone represses the SETD2-mediated H3K36 tri-methylation through tightening the DNA wrapping around core histones.

To further analyze whether the linker histone could inhibit the H3K36me3 in cells, we performed chromatin immunoprecipitation coupled with high-throughput sequencing (ChIP-Seq) by using H3K36me3 and H1.4 antibodies, respectively. The rabbit anti-mouse IgG was used as the negative control. Individual browser track views showed that the two replicates of each ChIP-Seq result were very similar (Fig. 3c). We calculated the read densities of each ChIP-Seq by a 10-bp window in the genome and then compared their correlations (Supplementary Fig. S5b). The two biological replicates of ChIP-Seq were highly consistent, further confirming that these ChIP-Seq results are repeatable and stable. Interestingly, we found that the correlations between H3K36me3 and H1.4 were very low, suggesting that H3K36me3 and H1.4 are distributed in different regions of the genome. To further compare the enrichments of H3K36me3 and H1.4, we called ChIP-Seq peaks and analyzed their overlap by combining the two replicates (Fig. 3d). The results showed that only around 1% of the called H3K36me3 peaks were overlapped with H1.4 peaks. More importantly, this low number of overlapped peaks was similar to that of the overlapped peaks between IgG control and other ChIP-Seq results. Supporting this, the H3K36me3 peaks were mainly distributed at exons and introns which were the gene body regions, and H1.4 peaks were enriched at intergenic regions and introns (Fig. 3e).

To gain insight into the chromatic incorporations of H3K36me3 and H1.4, we then compared the normalized density of ChIP-Seq reads at H3K36me3 and H1.4 peaks, respectively (Fig. 3f; Supplementary Fig. S5c). We found that H3K36me3 was barely detected at the H1.4 peaks where H1.4 was highly enriched. The reduction of the H1.4 signal was also detected at the H3K36me3 peaks, but with a lower level of reduction compared with that of H3K36me3 at H1.4 peaks. This may be because the H3K36me3 peaks were very broad and H1.4 could not be completely absent at these broad regions. We further compared the enrichment of H3K36me3 and H1.4 around the gene body regions where H3K36me3 was enriched (Fig. 3g). The H3K36me3 was enriched as a gradual increase from 5′ end to 3′ end of the gene body that is consistent with previous results^24^. H1.4 was enriched at the transcription starting site (TSS) where the H3K36me3 level was low. The results suggest that the distributions of H3K36me3 and H1.4 are mostly mutually exclusive in the chromatin. Still, future studies will be needed to determine whether this relationship is causal.

### Structural comparison with yeast Set2–xNCP^H3K36M^ complex

**Fig. 4:**
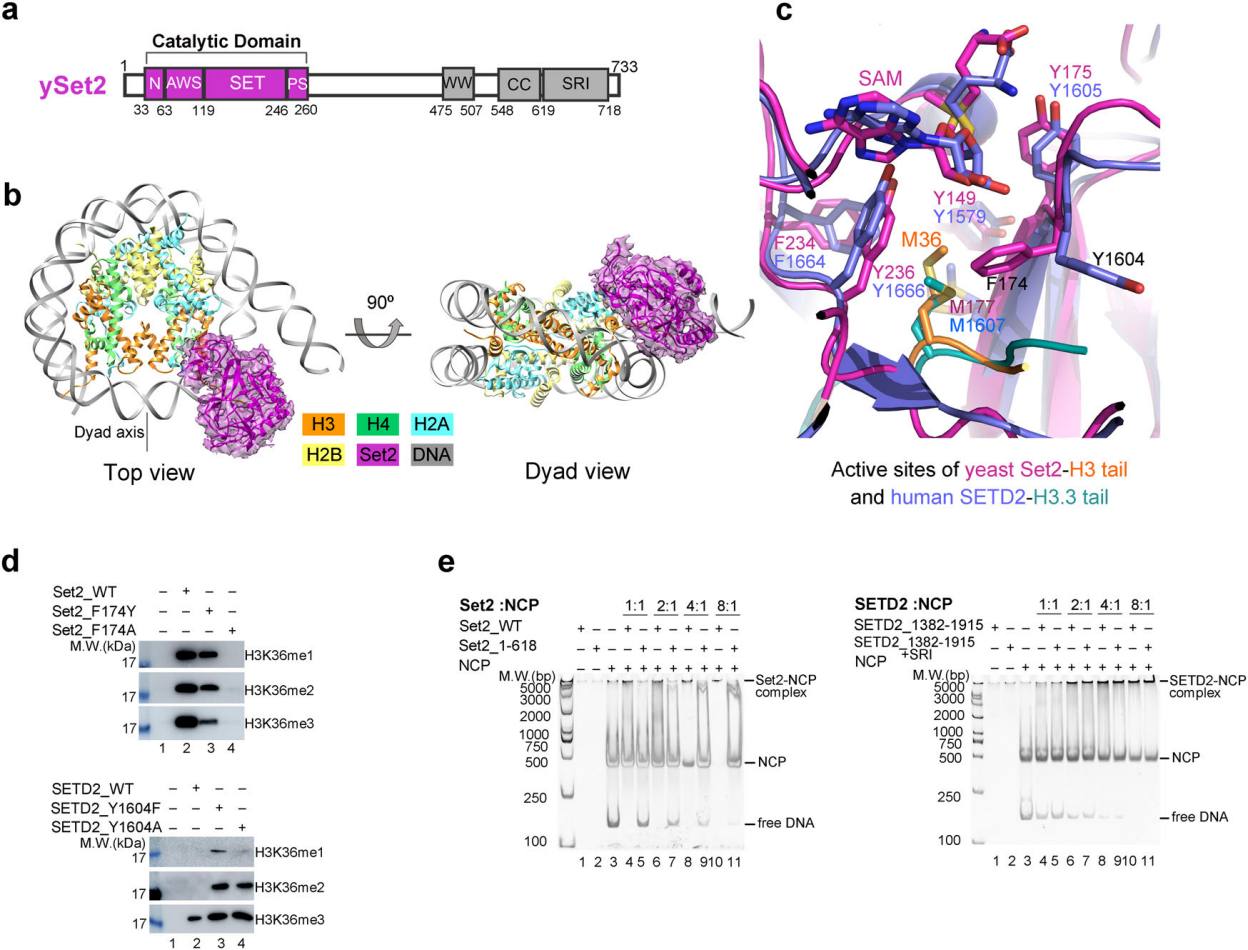
Structural and biochemical comparison between human SETD2–hNCP^H3.3K36M^ and yeast Set2–xNCP^H3K36M^ complexes. **a** Schematic drawing of the domain organizations of yeast Set2. The color scheme is the same as that of the Set2–xNCP^H3K36M^ structural model. **b** Cryo-EM density map and atomic model of yeast Set2–xNCP^H3K36M^ complex shown from two orthogonal views. The cryo-EM map was segmented and colored according to the respective components of the Set2–xNCP^H3K36M^ complex. **c** The detailed view of the active site in yeast Set2 and human SETD2. **d** HMT assays of wild-type and mutant Set2 (top) and SETD2 (bottom) proteins against nucleosome substrates. Mono-, di-, and tri-methylation levels of histone H3K36 are determined with antibodies of H3K36me1, H3K36me2, and H3K36me3. The input of the reactions is shown in Supplementary Fig. S10d. Each assay was repeated at least three times with similar results. **e** EMSAs of different truncations of Set2 (left) and SETD2 (right) with nucleosome at molar ratios of 1:1, 2:1, 4:1, and 8:1. The Native-PAGE gel is stained with ethidium bromide (EB) to show the gel shifting of NCP by Set2/SETD2. The input of the assays is shown in Supplementary Fig. S11d.

Set2 is the sole H3K36 methyltransferase that carries out mono-, di- and tri-methylation of H3K36 in yeast^31^. To understand the structural basis for nucleosome recognition by yeast Set2 and the underlying mechanism that determines the activity difference between yeast Set2 and human SETD2, we solved the cryo-EM structure of full-length yeast Set2 complexed with an H3K36M mutant *Xenopus laevis* nucleosome (hereafter, xNCP^H3K36M^) at an overall resolution of 3.3 Å (Fig. 4a, b; Supplementary Figs. S6, S7 and Table S1). In the yeast Set2–xNCP^H3K36M^ complex, the catalytic domain of Set2 interacts with nucleosome in a similar manner as the human SETD2–hNCP^H3.3K36M^ association and the fungal Set2–nucleosome interaction^30^ (Fig. 4b; Supplementary Fig. S3a). Nucleosome DNA unwrapping at SHL-6 and SHL-7 as well as the specific interaction between the α-N helix of histone H3 and the AWS domain of Set2 serve as the evolutionarily conserved structural features for the nucleosome binding by Set2 from yeast to human (Supplementary Fig. S8). In addition, cryo-EM analysis of yeast Set2 in complex with a *Xenopus laevis* wild-type nucleosome (hereafter, xNCP^WT^) also failed to reveal the EM density of Set2 on xNCP^WT^, consistent with our observations in human SETD2 studies (Supplementary Fig. S9).

Structural comparison at the active sites of yeast Set2 and human SETD2 revealed that the side chain of the K36M mutation sticks into a hydrophobic pocket of Set2 (composed of residues Tyr149, Phe174, Met177, Phe234, and Tyr236) that is mostly conserved with that of human SETD2 (Fig. 4c). However, a remarkable difference in the side-chain conformations of Phe174^Set2^ and its counterpart Tyr1604^SETD2^ was observed between the two structures (Fig. 4c; Supplementary Fig. S10a–c). The side chain of Tyr1604^SETD2^ has pointed away from the hydrophobic H3.3M36-binding pocket, whereas the aromatic ring of Phe174 at the active site of Set2 is oriented toward the side chain of Met36^H3^, shaping the H3K36-binding pocket (Fig. 4c; Supplementary Fig. S10a, b). Mutagenesis analysis revealed that substitution of Phe174^Set2^ with Tyrosine (F174Y) severely impaired the mono-, di- and tri-methylation of H3K36 and a Phenylalanine-to-Alanine substitution (F174A) completely abolished the activities of Set2, suggesting an essential role of Phe174 in the recognition of H3K36 in yeast Set2 (Fig. 4d; Supplementary Fig. S10d). By contrast, similar mutations of Tyr1604 (Y1604F and Y1604A) of human SETD2 remarkably boosted the activities of SETD2 and resulted in the di-methylation and a slight mono-methylation in addition to the H3K36 tri-methylation (Fig. 4d; Supplementary Fig. S10d). In a previous study, the Y1604A mutant of SETD2 also showed elevated activities, indicating the important role of Y1604 in regulating the activity of SETD2^25^. This observation implies that Tyr1604^SETD2^ might fine-tune the methyltransferase activity of SETD2 and also contribute to the determination of substrate specificity of SETD2. In addition, to investigate the effect of Phe1996 that corresponds to Tyr1604 in SETD2 on the methyltransferase activities of NSD1 (Supplementary Fig. S11a), an HMT activity assay was performed for further mutagenesis analysis. It was shown that the substitution of Phe1996^NSD1^ with Tyr (F1996Y) decreased the mono- and di-methylation of H3K36, and Phenylalanine-to-Alanine substitution (F1996A) more severely hampered the activities of NSD1 (Supplementary Fig. S11b). These results suggest an essential role of Tyr1604^SETD2^/Phe1996^NSD1^ in the methylation of H3K36.

Previous studies revealed that deletion of the SRI domain of Set2 (Set2^SRI^) abolished the interaction of Set2 with RNA pol II and impaired H3K36 methylation during transcription elongation in yeast^6^. The SRI domain was also reported to be required for the in vitro methyltransferase activity of Set2^5^, which was observed in our analysis, as well (Supplementary Fig. 11c). In addition, EMSA assays demonstrated that lack of the SRI domain severely impaired the formation of the Set2–xNCP^H3K36M^ complex, suggesting that Set2^SRI^ is involved in nucleosome binding (Fig. 4e, left panel; Supplementary Fig. S11d, left panel). However, Set2^SRI^ is not visible in the cryo-EM density map of the yeast Set2–xNCP^H3K36M^ complex, which implies that Set2^SRI^ might interact with nucleosome in a nonspecific way. In a similar analysis of the SRI domain of human SETD2 (SETD2^SRI^), although SETD2^SRI^ could result in a three-fold increase in the activity of the catalytic domain of SETD2 against nucleosomes, it did not appear to participate in the binding of the nucleosome (Fig. 4e, right panel; Supplementary Figs. S1b and S11d, right panel). These results suggest that the SRI domain might regulate the activities of Set2 and SETD2 in different ways.

## Discussion

**Fig. 5:**
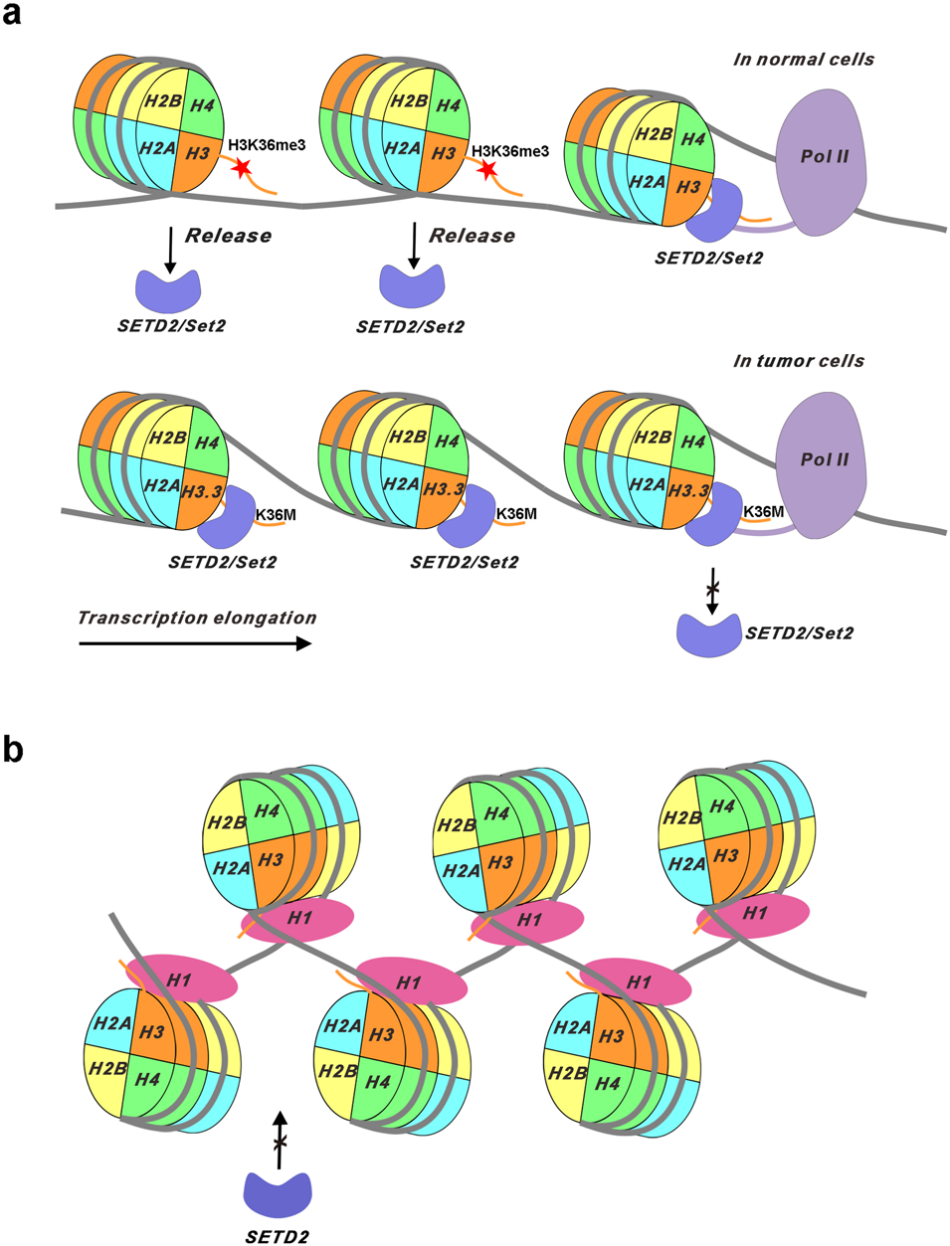
A working model for the actions of SETD2 on nucleosomes. **a** In normal cells, SETD2/Set2 directly associates with RNA Pol II and implements H3K36 methylation on partially unwrapped nucleosomes, co-transcriptionally. After that, SETD2/Set2 is rapidly released from the nucleosomes, and the DNA wraps back to its canonical conformation. However, in cancer cells carrying the oncogenic H3.3K36M mutations, SETD2/Set2 is withheld on the H3.3K36M nucleosome, which impairs the normal functions of SETD2/Set2 and possibly affects the movement of RNA pol II during transcription elongation. **b** A schematic drawing shows that histone H1 stabilizes the compact conformation of the nucleosome and prohibits the binding of SETD2 on the nucleosome.

In this work, the cryo-EM studies of human SETD2 methyltransferase complexed with an H3.3K36M mutant nucleosome or a wild-type nucleosome provide structural mechanisms for SETD2 catalysis as well as for inhibition of SETD2 by the oncogenic H3.3K36M mutation in the context of nucleosomes. We observed that the catalytic domain of SETD2 dynamically interacts with the wild-type nucleosome and the cofactor product *S*-adenosyl-l-homocysteine (SAH), suggestive of a transient binding mechanism for SETD2 and nucleosome. The ‘transient binding’ mechanism could allow rapid release of SETD2 from its nucleosome substrates, which might be suited to match the pol II-associated deposition of H3K36me3 marks with the transcription elongation rate (Fig. 5a). In contrast with the wild-type nucleosome, the H3.3K36M mutation stabilizes the complex of SETD2 with its nucleosome substrate and the cofactor SAM, resulting in the retention of SETD2 on nucleosome surface (Fig. 5a). Previous studies indicated that the H3.3K36M peptide binds to SETD2 in a more stable way than the wild-type H3.3K36 peptide does, and inhibits the activities of SETD2^24,26^. Our in vitro HMT assays further demonstrated that the H3.3K36M nucleosome exhibited stronger inhibitory effects on the activities of SETD2, compared with that of the H3.3K36M peptide (Supplementary Fig. S12). These observations collectively suggest that the H3.3K36M mutant nucleosome could potentially reduce the amounts of SETD2 that is available for H3K36 tri-methylation co-transcriptionally, and consequently impede the establishment of H3K36 methylation landscape and lead to the alteration of gene transcription. Future studies are required to further explore the mechanism in vivo.

The association of SETD2 with nucleosome requires the unwrapping of nucleosome DNA at its entry/exit site to expose the N-terminal region of histone H3 (Fig. 2a). Rapid DNA unwrapping and rewrapping, also called ‘DNA breathing’, results from the intrinsic structural dynamics of nucleosome^41^. It frequently occurs during the passage of RNA pol II, where SETD2 implements H3K36 tri-methylation co-transcriptionally. We also found that the linker histone H1 represses the methyltransferase activities of SETD2 toward nucleosomes and that it displays distinct distributions from the H3K36me3 marks in the genome (Fig. 3). Histone H1 binds the nucleosome linker DNA at the entry and exit sites, which stabilizes the compact conformation of the nucleosome and blocks the binding site of SETD2 on the nucleosome surface (Fig. 5b). Previous studies revealed that eviction of histone H1 from chromatin enhances gene transcription activation^42–44^. In addition to its effect on chromatin decondensation, the histone H1 displacement could also restore the dynamics of nucleosome conformation and favors H3K36 tri-methylation and other histone modifications accompanied with active transcription. Previous findings that H3.3 maintained a decondensed chromatin state and antagonizes H1 incorporation in the early development of mice also suggest mutually exclusive functions of histone H3.3 and H1 on chromatin regulation^45^.

The cryo-EM structures of human SETD2 and its yeast homolog Set2 complexed with the H3K36M nucleosome also provide an evolutionarily conserved structural framework for the recognition of nucleosome by the histone H3K36 methyltransferases. The AWS domains of SETD2 and Set2 specifically interact with the α-N helix of histone H3, where the side chains of Arg49 and Arg52 of H3 are inserted into a conserved concaved surface of AWS (Fig. 2b; Supplementary Fig. S8b). The R49E&R52E mutant nucleosome failed to be methylated by SETD2, Set2, and the H3K36 di-methyltransferase NSD1, highlighting the importance of this specific recognition in H3K36 methylation (Fig. 2c, d; Supplementary Fig. S8c). A similar interaction interface was also observed in the fungal Set2–nucleosome structure^30^, suggesting that it provides a common structural basis for H3K36 methylation. In addition, the recognition interfaces of the K36M mutation with the active site of SETD2 and Set2 are mostly similar except for the side-chain conformations of the Phe174^Set2^/Tyr1604^SETD2^ pair (Fig. 4c). The biochemical analysis further indicated that the two residues play opposite roles in the activity regulation of Set2 and SETD2, and Tyr1604^SETD2^ also participates in the determination of substrate specificity in SETD2 (Fig. 4d). A ‘Phe/Tyr switch’ mechanism has been previously proposed for controlling the methylation states of several SET-domain-containing HMTs^46,47^. Residues Y149 and F234 of Set2 were proposed to serve as the ‘Phe/Tyr switch’ in yeast^48^. It was shown that yeast Set2 bearing a Y149F mutation predominately catalyzes H3K36 tri-methylation, and an F234Y substitution results in the mono- and di-methylation of H3K36^48^. As human SETD2 and yeast Set2 have the identical residues (Tyr149^Set2^/Tyr1579^SETD2^, Phe234^Set2^/Phe1664^SETD2^) at the two positions (Supplementary Fig. S10c), the Phe174^Set2^/Tyr1604^SETD2^ pair identified in this study might serve as another type of ‘Phe/Tyr switch’ that accounts for the different methyltransferase activities between human SETD2 and yeast Set2.

In summary, our studies provide structural insights into the oncogenic effects of the H3.3K36M mutation on the molecular functions of the H3K36 methyltransferase SETD2 at the nucleosome level, and demonstrate the regulatory effects of nucleosome conformation dynamics on SETD2-mediated H3K36 tri-methylation. Comparative structural analysis of human SETD2 and yeast Set2 complexed with nucleosomes further revealed conserved structural features for the recognition of nucleosome and identified structural elements that account for the difference of the enzyme activities. The key residues and interaction interfaces revealed in this work may be targeted for further functional investigation of SETD2 and also for the mechanistic elucidation of SETD2-related tumorigenesis.

## Materials and methods

### Protein expression and purification

Human SETD2, yeast Set2, and their truncations or mutants were cloned into a modified pET28b vector with a 6× His-SUMO tag fused at the N-terminus. The proteins were individually expressed in *E. coli* Transetta (DE3) and were purified with Ni-NTA agarose beads (QIAGEN) in lysis buffer (50 mM Tris–HCl, pH 7.5, 500 mM NaCl, 10% glycerol, 5 mM 2-mercaptoethanol, 1 mM PMSF, 5 mM benzamidine, 1 µg/mL leupeptin, and 1 µg/mL pepstatin). The 6× His-SUMO tag was removed through Ulp1 protease digestion and the proteins were further purified sequentially through Mono S 10/100 GL cation exchange chromatography (GE Healthcare) in Column Buffer A (25 mM MES, pH 6.0) and column buffer B (25 mM MES, pH 6.0 and 1 M NaCl) and HiLoad Superdex 200 gel filtration chromatography (GE Healthcare) in column buffer C (25 mM Tris–HCl, pH 7.5 and 150 mM NaCl). The purified proteins were concentrated to 5 mg/mL and stored at –80 °C in small aliquots.

### Preparation of nucleosomes

Wild-type or H3/H3.3K36M-mutant nucleosomes were in vitro reconstituted from four core histones (H3/H3.3 or H3/H3.3K36M, H4, H2A, and H2B) and the 147-bp or 197-bp Widom 601 DNA as previously described^49^. The human or *Xenopus laevis* histones and their mutants were expressed in *E. coli* BL21 (DE3) as inclusion bodies and were purified through Q Sepharose HP chromatography (GE Healthcare) and SP Sepharose HP chromatography (GE Healthcare) in denaturing buffer (20 mM Tris–HCl, pH 7.5, 8 M urea, 0–0.5 M NaCl, 1 mM EDTA, and 5 mM 2-mercaptoethanol), sequentially. After dialysis and lyophilization, the histone proteins were assembled into histone octamers and were purified on a HiLoad Superdex 200 gel filtration column in refolding buffer (20 mM Tris–HCl, pH 7.5, 2 M NaCl, 1 mM EDTA, and 5 mM 2-mercaptoethanol). Then, the histone octamers were mixed with the Widom 601 DNA at a molar ratio of 0.9:1.0, and the mixture was dialyzed against reconstitution buffer (10 mM Tris–HCl, pH 7.5, 1 mM EDTA, 1 mM DTT, and 0.15–2 M KCl). The reconstituted nucleosomes were further purified through HiLoad Superdex 200 gel filtration chromatography and were then stored in small aliquots at –80 °C.

### H1 deposition mediated by NAP-1

Human NAP-1 and H1.4_1–130aa truncation were cloned into a modified pETDuet vector with or without a 6× His tag fused at the N-terminus, respectively. The proteins were individually expressed in *E. coli* BL21 (DE3) cells as previously described^50^. His-NAP-1 was purified from the supernatant of the bacterial lysate using Ni-NTA agarose beads, followed by Mono Q 10/100 GL anion exchange chromatography (GE Healthcare). H1.4_1–130aa was purified through Mono Q 10/100 GL anion exchange chromatography (GE Healthcare) and Mono S 10/100 GL cation exchange chromatography, sequentially.

For H1 deposition, the NAP-1 and H1.4 proteins were mixed at a molar ratio of 2:1 in the buffer of 20 mM Tris–HCl, pH 7.5, 0.5 mM EDTA, 100 mM NaCl, 1 mM DTT, 10% glycerol, and 0.1 mM PMSF, and were incubated at 30 °C for 15 min. Then, an equimolar ratio of nucleosomes and linker histone/NAP-1 complexes were mixed and further incubated at 30 °C for 30 min. His-tagged NAP-1 was removed by passing through Ni-NTA beads, and the H1–nucleosomes were concentrated to 5 mg/mL and stored at –80 °C in small aliquots.

### Sample preparation and cryo-EM data collection

The SETD2 or Set2 proteins were mixed with the wild-type or K36M-mutant NCPs and the cofactor SAH or SAM at a molar ratio of 8:1:10 and were incubated at 4 °C for 30 min. Then, the SETD2–hNCP^H3.3K36M^, Set2–xNCP^H3K36M^, SETD2–hNCP^WT^, or Set2–xNCP^WT^ complexes were purified using the method of GraFix^51^. In brief, a continuous 10%–30% gradient of glycerol with a 0–0.1% gradient of glutaraldehyde was generated on a BioComp gradient master. Then, the SETD2–hNCP^H3.3K36M^, Set2–xNCP^H3K36M^, SETD2–hNCP^WT^ or Set2–xNCP^WT^ mixture was loaded on top of the glycerol gradient and the ultracentrifugation was performed at the speed of 35,000 rpm at 4 °C for 15 h, using an SW41Ti or SW60Ti rotor (Beckman). The GraFix fractions containing the SETD2–hNCP^H3.3K36M^, Set2–xNCP^H3K36M^, SETD2–hNCP^WT^, or Set2–xNCP^WT^ complex were collected and dialyzed against 25 mM Tris–HCl, pH 7.5, and 150 mM NaCl, and were concentrated to 1 mg/mL. 2 µL aliquots of the samples were applied to glow-discharged holey carbon grids (Quantifoil R1.2/1.3, 300 mesh), and the grids were blotted for 2.5 s and were then plunged into liquid ethane cooled by liquid nitrogen, using a Vitrobot Mark IV (FEI).

The samples were observed under a Titan Krios transmission electron microscope (FEI) operated at 300 kV. The images were collected on a K3 direct electron detector (Gatan) with a pixel size of 1.09 or 1.10 Å. The SETD2–hNCP^H3.3K36M^, Set2–xNCP^H3K36M^, and Set2–xNCP^WT^ datasets have a defocus range of –0.7 to –2.8, –0.8 to –3.3, and –0.8 to –2.6 µm, respectively; and each micrograph was dose-fractioned to 32 frames with 0.1 s exposure time for each frame. The total accumulated dose of each micrograph is 50.0 e^−^/Å^2^. The dataset of the SETD2–hNCP^WT^ complex collected on a Falcon 3EC detector has a defocus range of –1.0 to –2.0 µm, and each micrograph was dose-fractioned to 38 frames with 1.3 s exposure time for each frame. The total accumulated dose of each micrograph is 40.0 e^−^/Å^2^. The imaging conditions were also listed in Supplementary Table S1.

### Image processing and model building

A total of 3931 cryo-EM images of the SETD2–hNCP^H3.3K36M^ complex were collected on a K3 detector, and motion correction was performed on the dose-fractioned image stacks using MotionCor2 with dose weighting^52,53^. The CTF parameters of each image were determined with Gctf^54^. Particle picking, 2D classification, 3D initial model, 3D classification, 3D auto-refine, CTF refinement, and Bayesian polishing were performed with RELION-3^55^. An overview of the data processing procedure was shown in Supplementary Fig. S2a. After two rounds of 2D classification and two rounds of 3D classification with exhaustive angular searches, a total of 339,177 particles that belong to the SETD2–hNCP^H3.3K36M^ complex were processed with 3D auto-refine and solvent-masked post-processing. To improve the map density of SETD2, the particles were further processed through masked 3D classifications with partial signal subtraction^56^, and a cryo-EM map of the SETD2–hNCP^H3.3K36M^ complex was finally calculated from 154,984 particles at an overall resolution of 3.1 Å. The resolution estimation was based on the gold-standard Fourier shell correlation (FSC) 0.143 criterion and the local resolution was estimated with ResMap^57,58^. The cryo-EM datasets of the Set2–xNCP^H3K36M^, SETD2–hNCP^WT^, and Set2–xNCP^WT^ complexes were processed similarly to that of the SETD2–hNCP^H3.3K36M^ complex.

Model building was carried out by fitting the available structures of NCP and human SETD2 (PDB codes: 5X7X, 6J99, and 5JJY) in the EM density maps of the SETD2–hNCP^H3.3K36M^ and Set2–xNCP^H3K36M^ complexes using UCSF Chimera^59^. The model was then manually built in Coot and real-space refined with secondary structure restraints in Phenix^60,61^.

### HMT assay

For an 8-µL HMT reaction, 0.2 µM wild-type or mutant SETD2 and Set2 proteins, 20 µM SAM, and 2 µM NCP were mixed in the buffer of 50 mM Tris–HCl, pH 8.0, 2 mM DTT, 5% glycerol, and 0.4 mg/mL BSA, and were incubated at 30 °C for 2 and 3 h, respectively. The reaction was stopped by adding 2 μL of 0.5% trifluoroacetic acid (TFA) and the HMT activity was evaluated using an MTase-Glo^TM^ Methyltransferases Assay Kit (Promega). The luminescent signal that corresponds to the production of SAH was measured using the EnSpire Alpha Multimode plate reader (PerkinElmer) in a white 384-well plate. Each reaction was run in triplicate and was reported as the means ± standard deviation (SD).

For evaluation of the activity specificity of SETD2 and Set2, the HMT reaction mixtures were separated on a 15% SDS–PAGE gel, and the mono-, di- and tri-methylation of histone H3K36 was detected by western blotting using the corresponding antibodies (H3K36me1 antibody: #A2364, ABclonal; H3K36me2 antibody: #A2365, ABclonal; H3K36me3 antibody: #A2366, ABclonal).

### EMSA

SETD2 or Set2 was mixed with 1 µM NCP^WT^ or NCP^H3K36M^ at a molar ratio of 1:1, 2:1, 4:1, or 8:1 in a total volume of 20 µL. After incubation on ice for 1 h, 10 µL of each mixture was run on a 6% Native-PAGE gel and the gel was stained with EB to show the shift of NCP^WT^ or NCP^H3K36M^. The same amount of each mixture was loaded on a 13.5% SDS–PAGE gel and detected by Coomassie blue staining to show the input of the EMSA assays.

### ChIP-Seq and data analysis

ChIP-Seq was conducted as described before with modifications described below^62^. Cells were cross-linked with 1% formaldehyde and quenched in 125 mM glycine. Nuclei were extracted by lysis buffer (10 mM Tris–HCl, pH 7.5, 10 mM NaCl, 0.5% NP-40), and then digested in MNase digestion buffer (20 mM Tris–HCl, pH 7.5, 15 mM NaCl, 60 mM KCl, 1 mM CaCl_2_) with MNase at 37 °C for 20 min. After mixing with equal amounts of 2× STOP buffer (100 mM Tris–HCl, pH 8.1, 20 mM EDTA, 200 mM NaCl, 2% Triton X-100, 0.2% sodium deoxycholate), the digested nuclei were sonicated for 5 min with biorupter (30 s on/30 s off). The extracted chromatin was incubated with 2 μg of anti-H1.4 (Cell Signaling Technology, Cat. #41328), anti-H3K36me3 (Active Motif, Cat. #61101) antibodies, and rabbit anti-mouse IgG (Abcam, Cat. #ab6709) at 4 °C overnight, respectively. The antibody-bound chromatin was then incubated with 30 μL of protein G-magnetic beads (Life Technologies) for 2 h. The beads were extensively washed before the release of enriched DNAs. DNA libraries were prepared by TruPrep DNA library prep kit (Vazyme) and subsequently sequenced on the Illumina HiSeq 4000 platform.

ChIP-Seq reads were cleaned by trim-galore and then aligned to the human genome, hg19, by bowtie2^63^. The duplicated PCR reads were further cleaned by SAMtools^64^. The H3K36me3 and H1.4 ChIP-Seq peaks were identified by MACS2^65^ with the parameters of the broad peak for H3K36me3, and the narrow peak calling for H1.4. BEDTools^66^ and deeptools^67^ were used to calculate the read density at different regions.

## Supplementary information

Supplementary Information

## Data Availability

The EM density maps have been deposited in the Electron Microscopy Data Bank with accession codes EMD-31039, EMD-31040, EMD-31041, and EMD-31042. The final models have been submitted to the RCSB Protein Data Bank under the accession codes 7EA5 and 7EA8. ChIP-Seq data have been submitted to the GEO database with GEO number GSE148235. All other data are available from the corresponding author upon reasonable request.
