# Supplementary material for: Cryo-EM structure of SETD2/Set2 methyltransferase bound to a nucleosome containing oncohistone mutations
Source: Cell Discov. 2021 May 11;7:32. doi: 10.1038/s41421-021-00261-6 (PMC8110526; doi:10.1038/s41421-021-00261-6)
Supplement: Supplementary file 1 — Supplementary Information [file 41421_2021_261_MOESM1_ESM.pdf]

Supplementary information for

Cryo-EM structure of SETD2/Set2 methyltransferase bound to a nucleosome containing oncohistone mutations

Authors: Yingying Liu[1,2,3], Yanjun Zhang[4], Han Xue[5], Mi Cao[1,2], Guohui Bai[1,2,3], Zongkai Mu[1,2,3], Yanli Yao[6,7,8], Shuyang Sun[6,7,8], Dong Fang[4]* and Jing Huang[1,2,3,7,9]*

Correspondence to: huangjing@shsmu.edu.cn and dfang@zju.edu.cn

This file includes:

Figures S1 to S12
Table S1

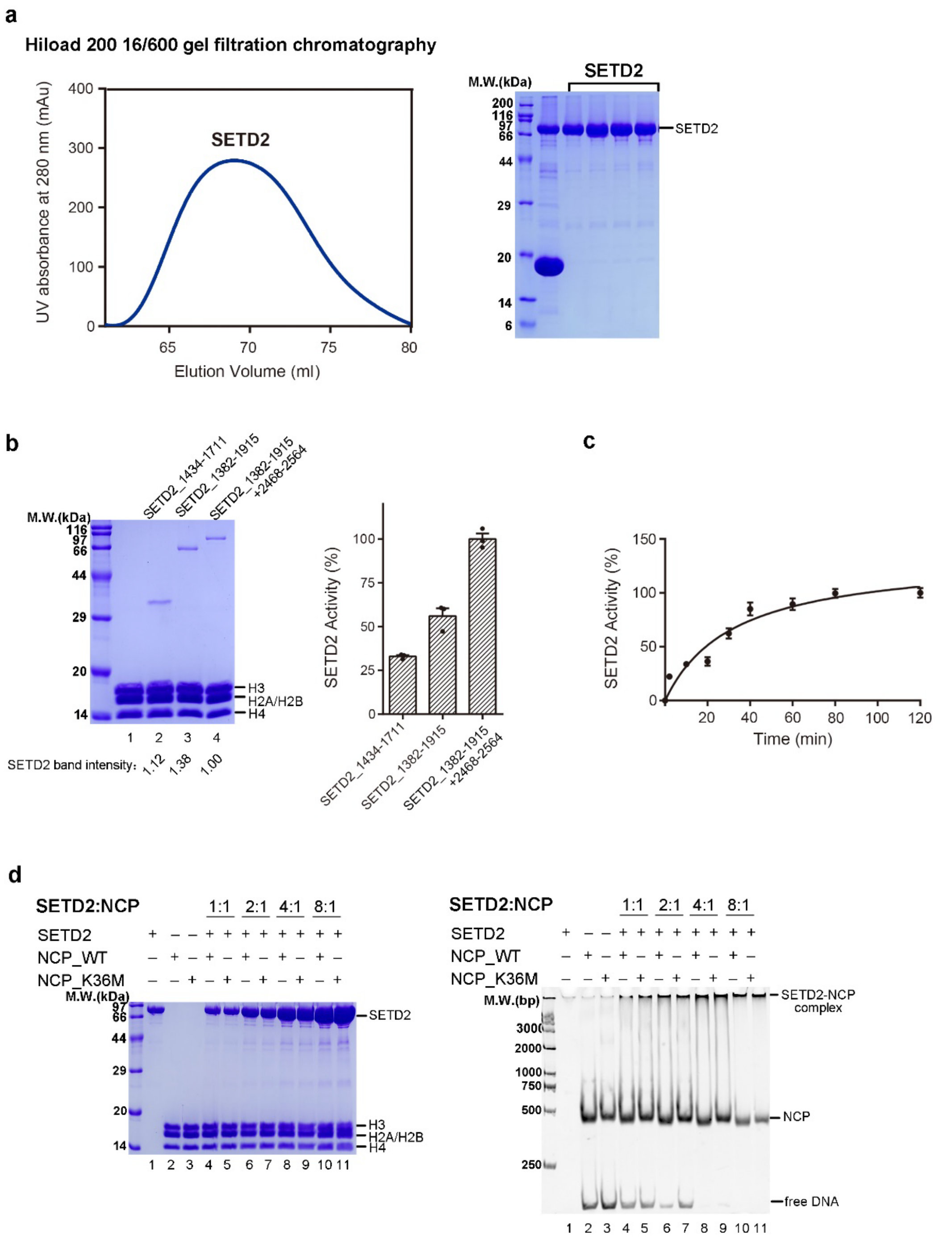

Supplementary Fig. S1. Biochemical characterizations of the SETD2-hNCP^{WT} and SETD2-hNCP$^{H3.3K36M}$ complexes. a, Gel filtration (left panel) and SDS-PAGE analysis (right panel) of the purification of human SETD2$^{1382\text{-}1915+2468\text{-}2564}$ proteins. **b**, Endpoint HMT assays performed with different truncations of SETD2. Left panel, input of the HMT reactions. The input amounts were quantified according to the band intensities of SETD2. Right panel, endpoint HMT assays of the different truncations of SETD2 against wild-type human NCPs. Error bars denote the standard deviation from the mean of three replicates. **c,** Time course of the H3.3K36 methylation catalyzed by SETD2$^{1382\text{-}1915+2468\text{-}2564}$ on wild-type hNCPs. Error bars denote the standard deviation

from the mean of three replicates. **d**, Electrophoretic mobility shift assays (EMSAs) of the SETD2 proteins with either wide-type or H3.3K36M mutant NCPs at molar ratios of 1:1, 2:1, 4:1, and 8:1. Left panel, input of the EMSA mixtures; right panel, Native-PAGE analysis of the gel shifting of hNCP and $hNCP^{H3.3K36M}$ by SETD2.

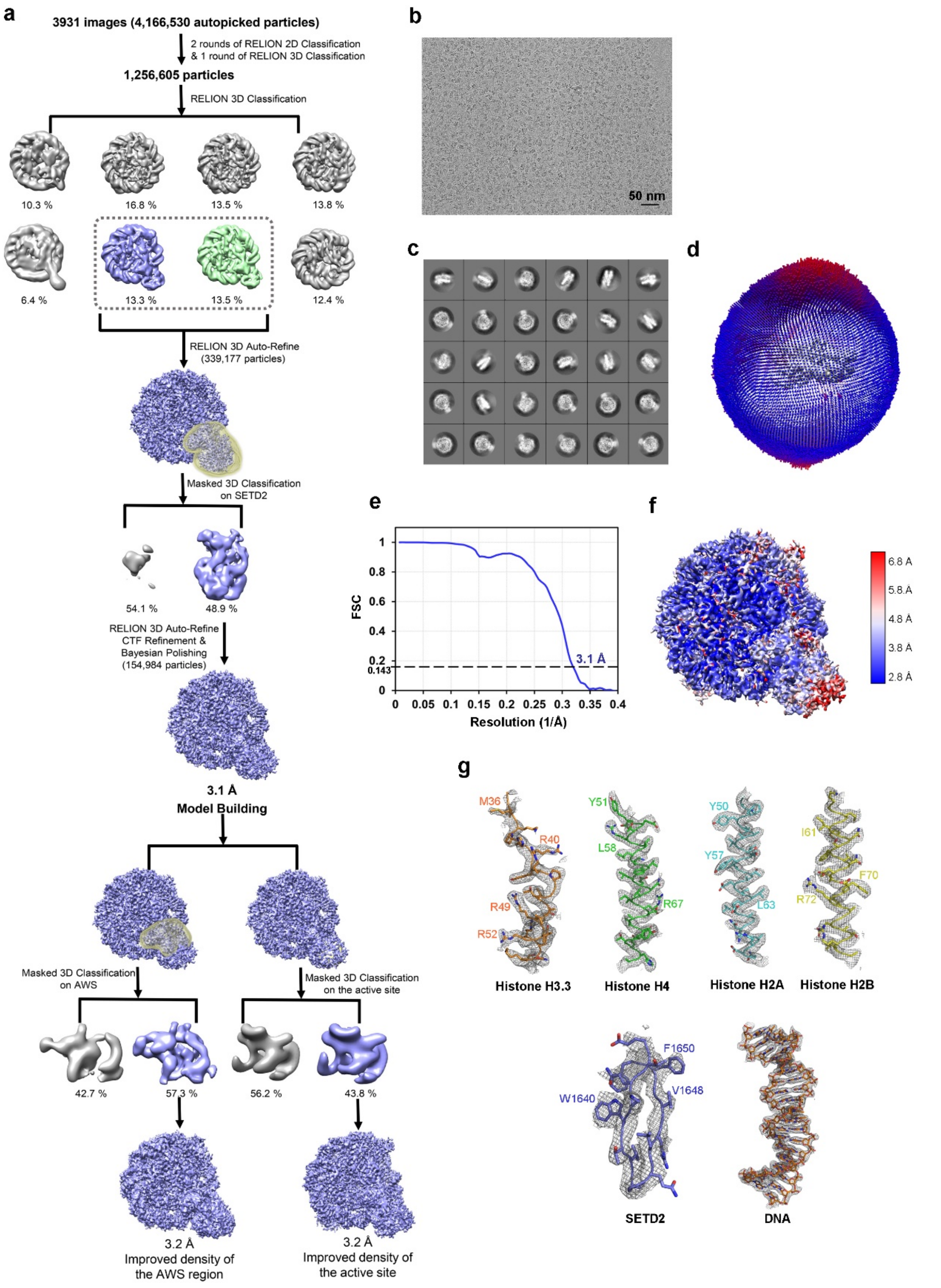

Supplementary Fig. S2. Cryo-EM analysis of human SETD2-hNCP$^{H3.3K36M}$ complex. a, Flow chart of cryo-EM data processing of the SETD2-hNCP$^{H3.3K36M}$ dataset (resolution: 3.1 Å). Masked 3D classifications without realignment were applied to the AWS domain (left branch) and the active site (right branch) of SETD2, respectively. **b**, Representative micrograph of the cryo-EM dataset of the SETD2-hNCP$^{H3.3K36M}$ complex. **c**, Representative 2D class averages of cryo-EM particles of the

SETD2-hNCP$^{H3.3K36M}$ complex. **d**, Angular distribution of particle projections of the SETD2-hNCP$^{H3.3K36M}$ reconstruction. **e**, The 'gold-standard' FSC curve calculated between two halves of the SETD2-hNCP$^{H3.3K36M}$ dataset. **f**, Local resolution estimates of the SETD2-hNCP$^{H3.3K36M}$ structure. **g**, Representative EM density maps of the SETD2-hNCP$^{H3.3K36M}$ complex.

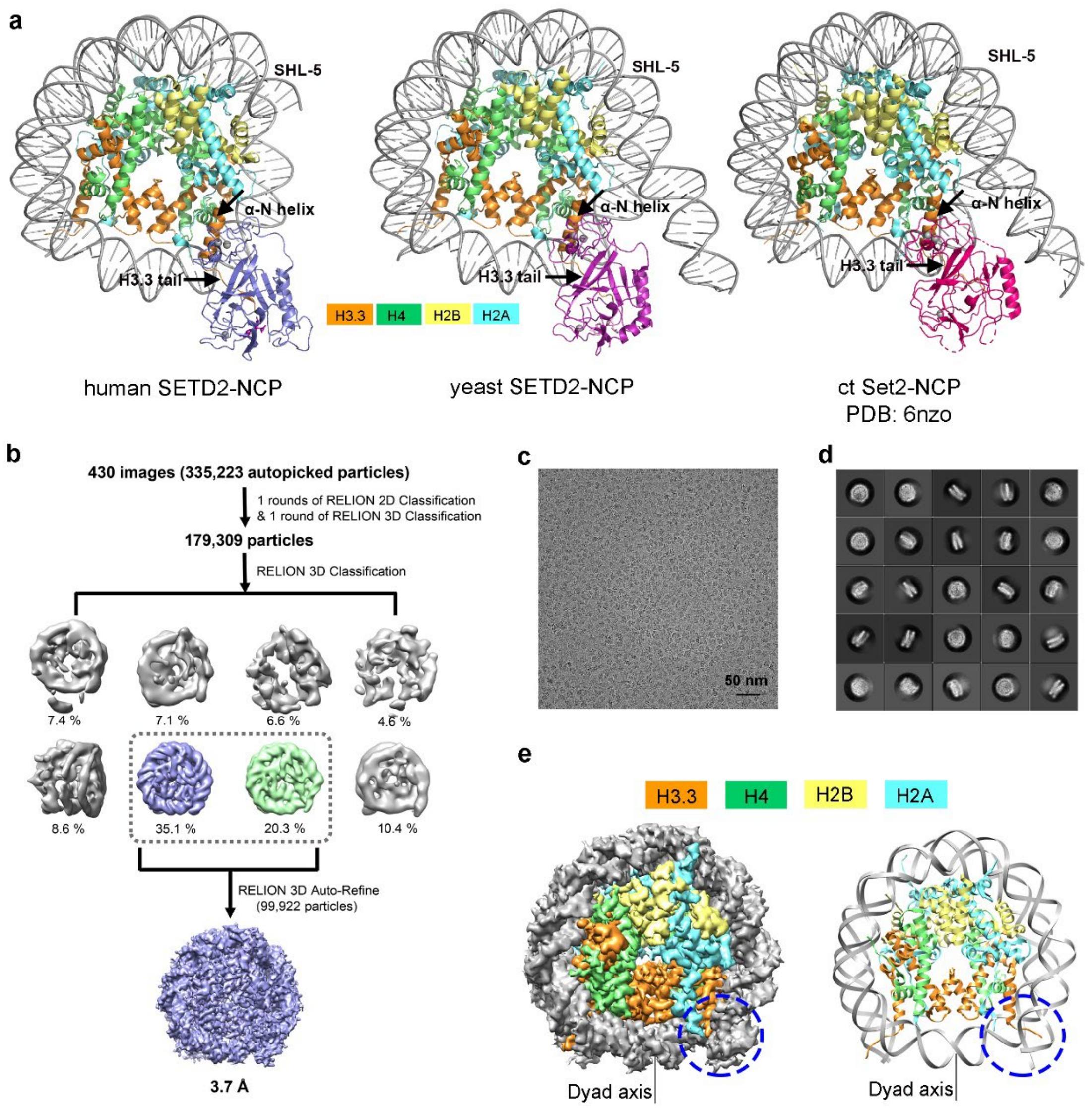

Supplementary Fig. S3. Structural comparation among SETD2 family proteins and cryo-EM analysis of human SETD2-hNCP^{WT} complex. a, Structural comparison of human SETD2-hNCP$^{H3.3K36M}$, yeast Set2-xNCP^{H3K36M} and *C. thermophilum* Set2- xNCP^{H3K36M} (PDB:6nzo) revealed evolutionarily conserved structural features for binding nucleosome by SETD2/Set2 in eukaryotes. **b**, Flow chart of cryo-EM data processing of the SETD2-hNCP^{WT} dataset (resolution: 3.7 Å). **c**, Representative micrograph of the cryo-EM dataset of the SETD2-hNCP^{WT} complex. **d**, Representative 2D class averages of cryo-EM particles of the SETD2-hNCP^{WT} complex. **e**, Cryo-EM density map (left panel) and atomic model (right panel) of the SETD2-hNCP^{WT} complex. The cryo-EM map was segmented and colored according to the respective components of the SETD2-hNCP^{WT} complex. SETD2 could not be observed on the wild-type nucleosome, and the potential SETD2-binding site was denoted with a dashed circle.

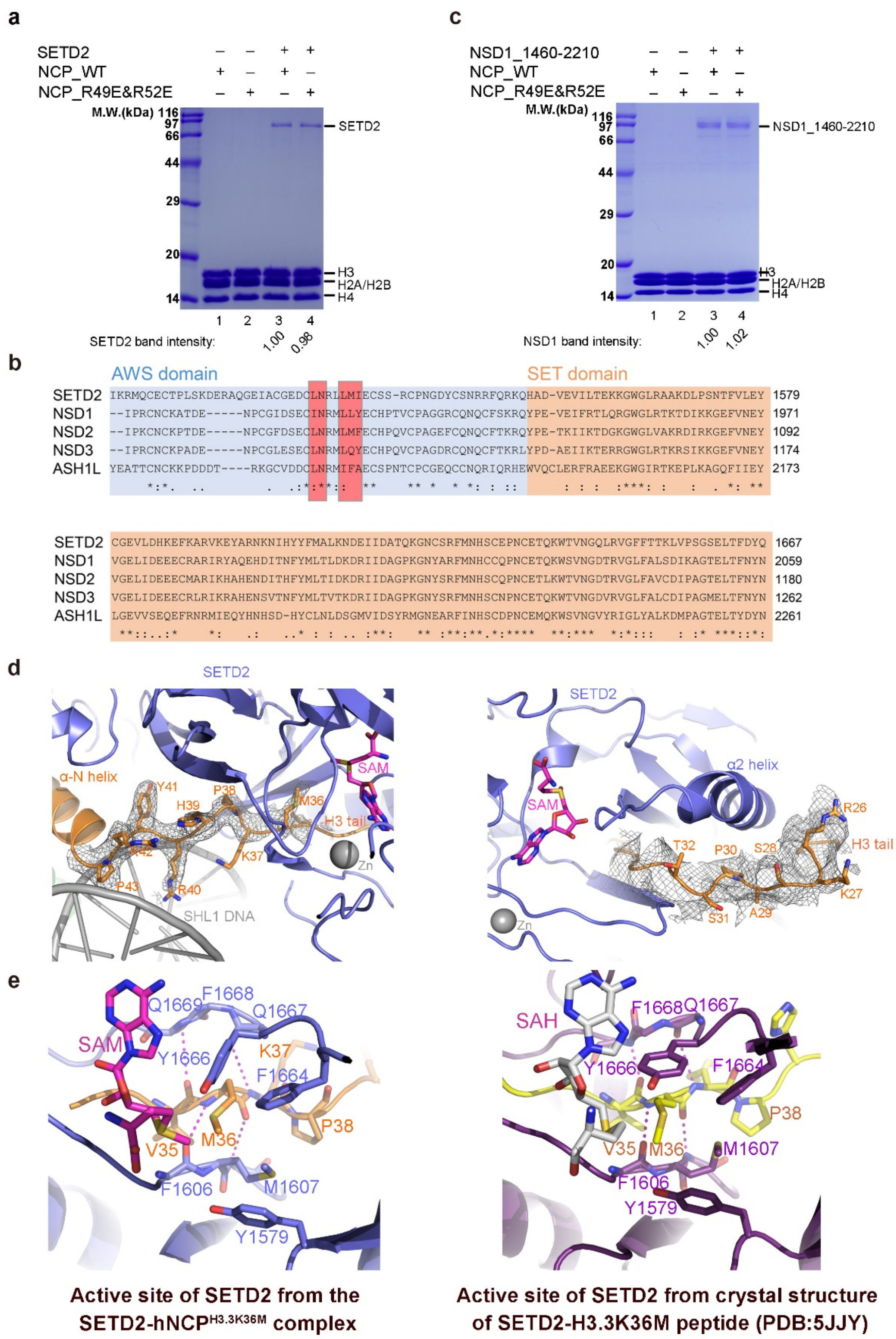

Supplementary Fig. S4. Detailed interactions between SETD2 and the H3.3K36M nucleosome. a, Input of the HMT reactions of SETD2 related to Fig. 2c. The input amounts were quantified according to the band intensities of SETD2. **b**, Sequence alignment of the AWS and SET domains of human histone H3K36 methyltransferases. The conserved residues for specific recognition of nucleosome are boxed in red. **c**, Input of the HMT reactions of NSD1 related to Fig. 2d. The input amounts were quantified

according to the band intensities of NSD1. **d**, The EM density map of the histone H3.3 tail in the SETD2-hNCP$^{H3.3K36M}$ complex structure. **e**, Structural comparison of the active site of SETD2 from the SETD2-hNCP$^{H3.3K36M}$ complex (left) and the SETD2-H3.3K36M peptide complex (right， PDB:5JJY). The active center of SETD2-nucleosome structure resembles that observed in the crystal structures of SETD2 complexed an H3.3K36M peptide.

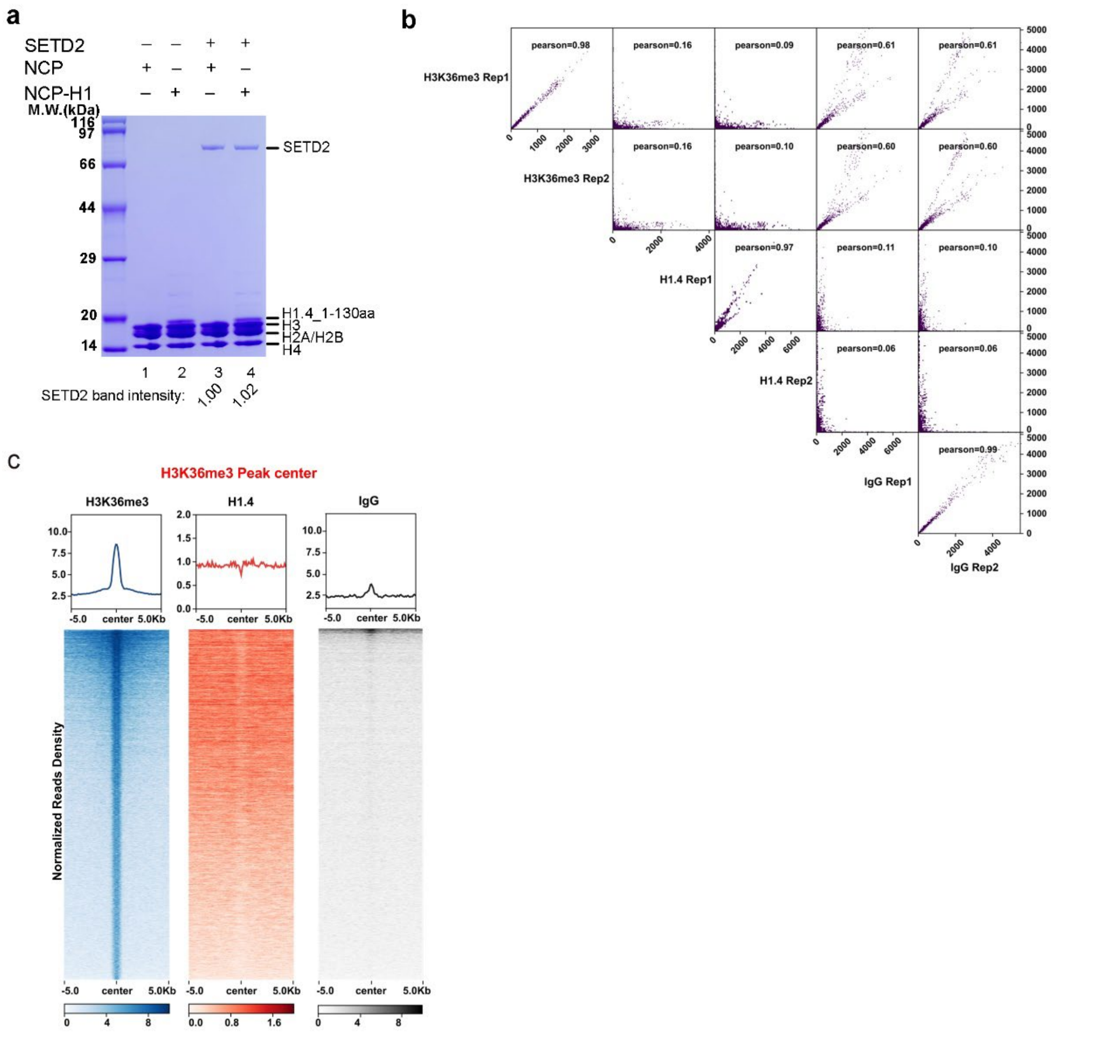

Supplementary Fig. S5. H3K36me3 and H1.4 are enriched at different regions across the genome. a, Input of the HMT reactions related to Fig. 3b, the input amounts were quantified according to the band intensities of SETD2. **b**, Scatter plot showing the correlations of H3K36me3, H1.4 and IgG across the genome. A 100 bp sliding window was used to scan the whole genome. **c**, The enrichments of H3K36me3, H1.4 and IgG ChIP-seq at H3K36me3 peak regions. A 100 bp sliding window was used to scan 10 kb regions surrounding the peak center. Heatmaps illustrating ChIP-seq reads densities from 5 kb upstream to 5 kb downstream of the peak regions (in rows) were shown on a per-peak basis (in columns).

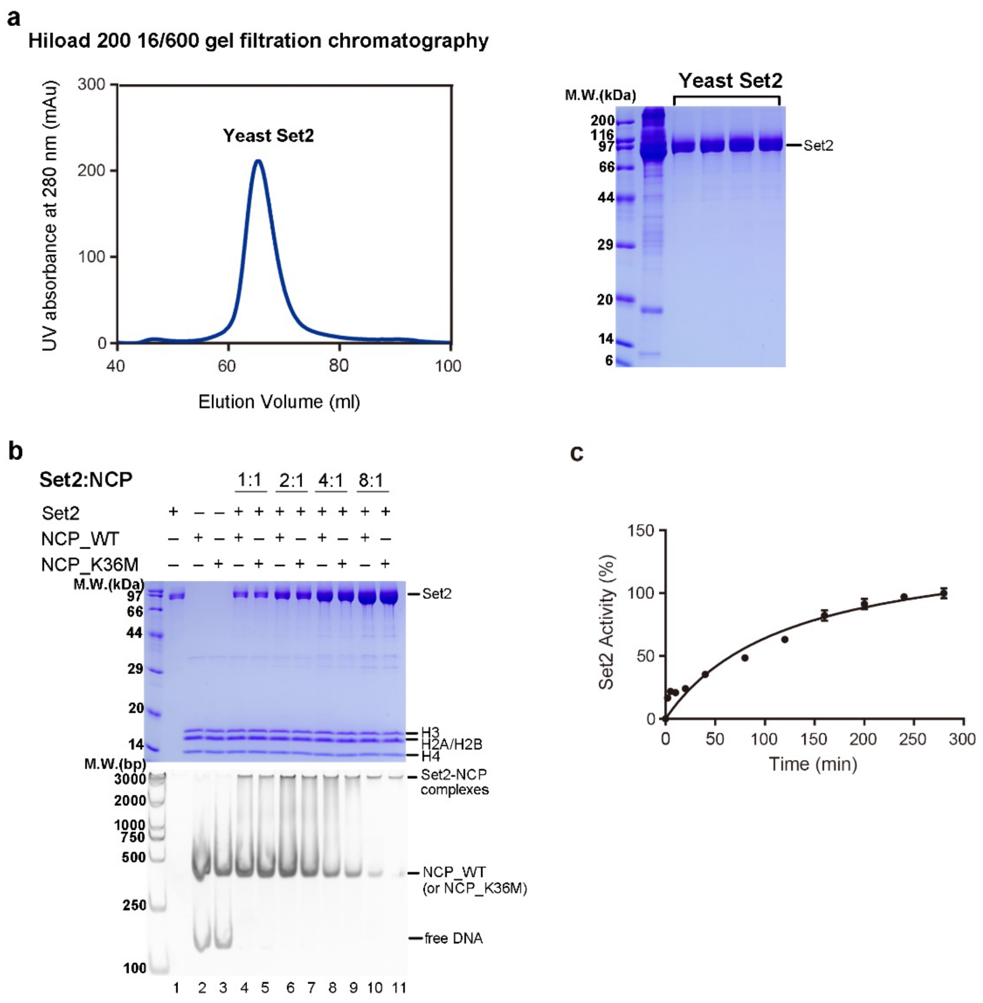

Supplementary Fig. S6. Biochemical characterizations of the Set2-xNCP^{WT} and Set2-xNCP^{H3K36M} complexes. a, Gel filtration (left panel) and SDS-PAGE analysis (right panel) of the purification of yeast Set2 proteins. **b**, Electrophoretic mobility shift assays (EMSAs) of the Set2 proteins with either wide type or H3K36M mutant xNCPs at molar ratios of 1:1, 2:1, 4:1, and 8:1. Upper panel, input of the EMSA mixtures; lower panel, Native-PAGE analysis of the gel shifting of xNCP and xNCP^{H3K36M} by Set2. **c**, Time course of the H3K36 methylation catalyzed by Set2 on wild-type xNCPs. Error bars denote the standard deviation from the mean of three replicates.

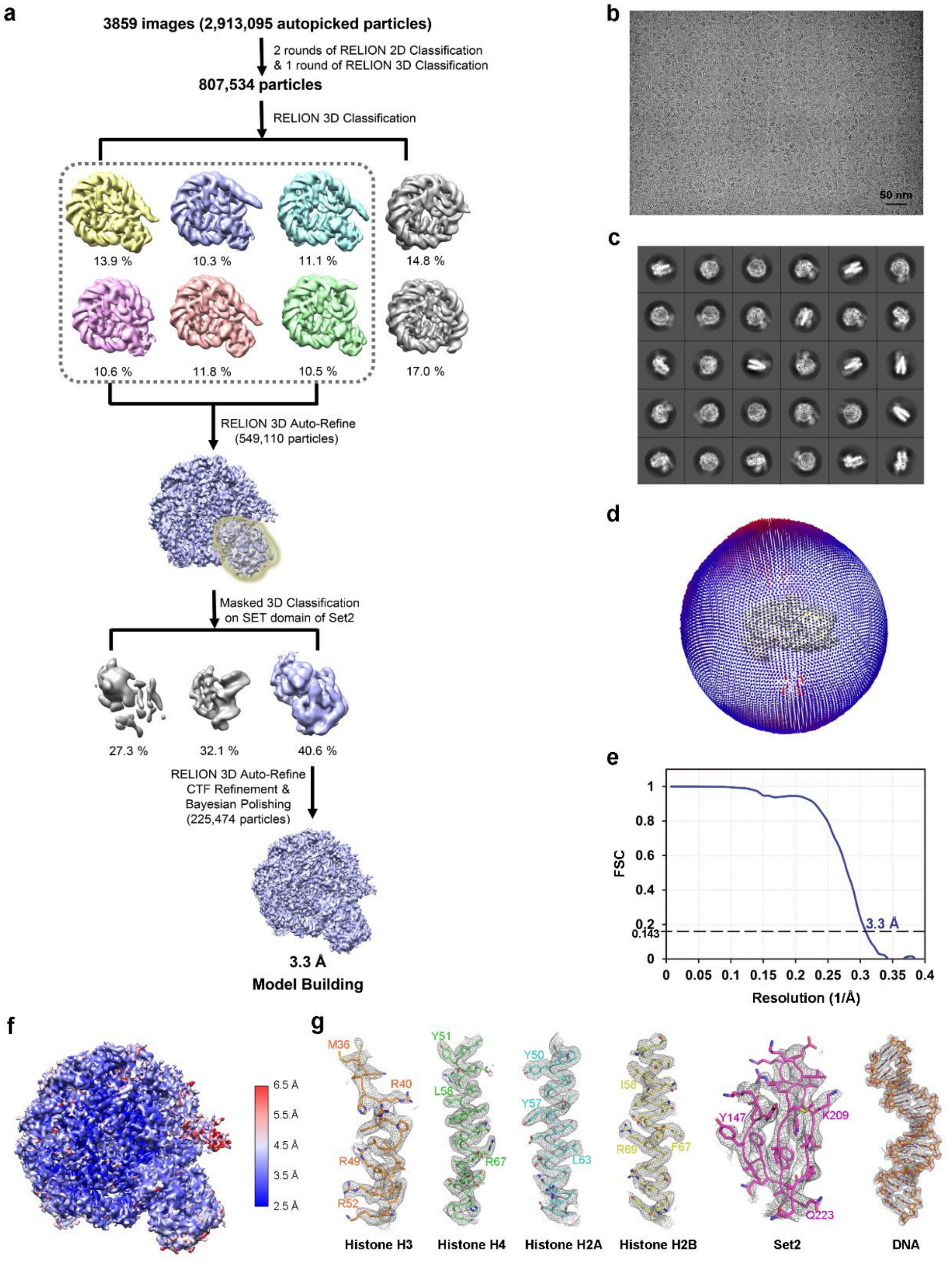

Supplementary Fig. S7. Cryo-EM analysis of yeast Set2-xNCP^{H3K36M} complex. a, Flow chart of cryo-EM data processing of the Set2-xNCP^{H3K36M} dataset (resolution: 3.3 Å). Masked 3D classification without realignment was applied to the catalytic domain of Set2. **b**, Representative micrograph of the cryo-EM dataset of the Set2-xNCP^{H3K36M} complex. **c**, Representative 2D class averages of cryo-EM particles of the Set2-xNCP^{H3K36M} complex. **d**, Angular distribution of particle projections of the Set2-xNCP^{H3K36M} reconstruction. **e**, The ‘gold-standard’ FSC curve calculated between two halves of the Set2-xNCP^{H3K36M} dataset. **f**, Local resolution estimates of the Set2-

xNCP^{H3K36M} complex structure. **g**, Representative EM density maps of the Set2-xNCP^{H3K36M} structure.

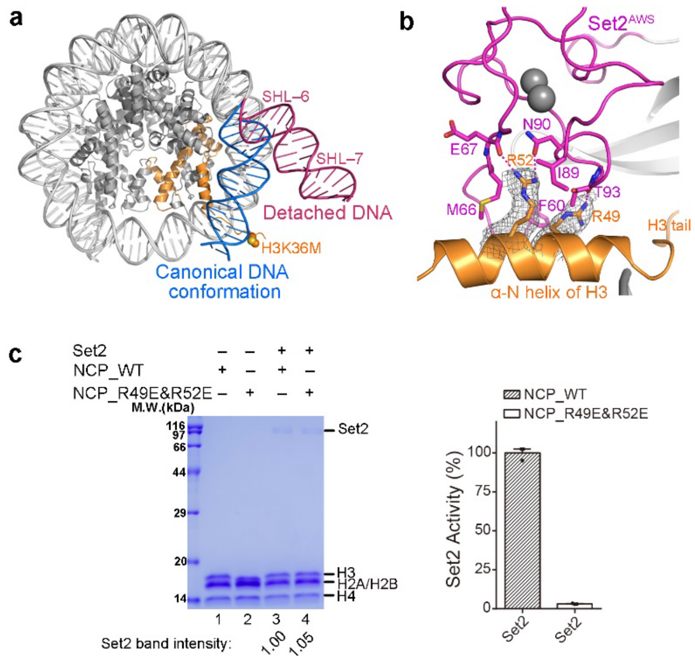

Supplementary Fig. S8. Specific recognition between Set2 and nucleosome. a, Nucleosome DNA at SHL-6 and SHL-7 is detached from the core histones in the Set2-xNCP^{H3K36M} complex structure. **b**, Detailed interactions between the AWS domain of Set2 and the α-N helix of histone H3. **c**, Endpoint HMT assays of Set2 against wild type and R49E&R52E-mutant xNCPs. Left panel, input of the HMT reactions. The input amounts were quantified according to the band intensities of Set2. Right panel, endpoint HMT assays of Set2 against wild type and R49E&R52E-mutant xNCPs. Error bars denote the standard deviation from the mean of three replicates.

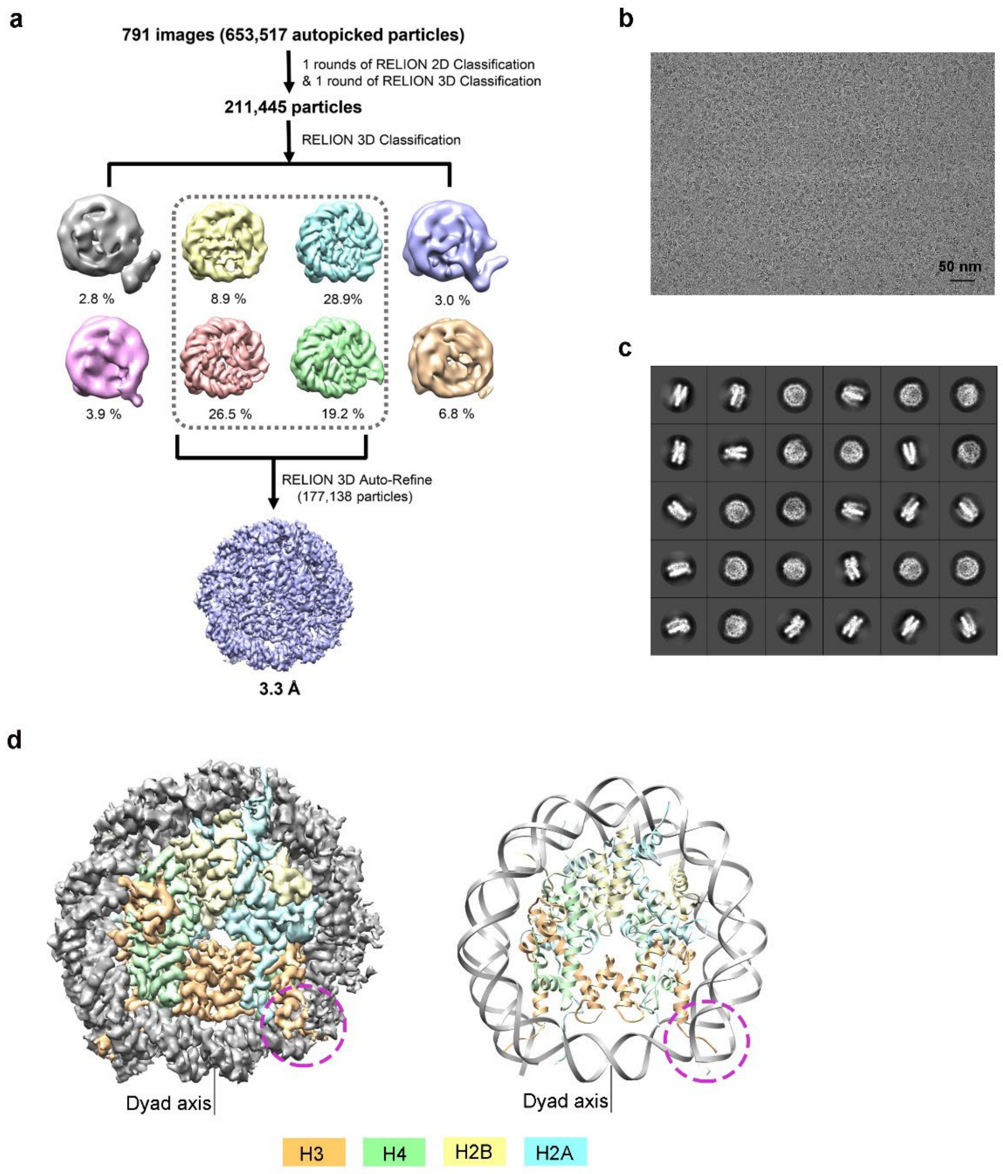

Supplementary Fig. S9. Cryo-EM data analysis of yeast Set2-xNCP^{WT} complex. a, Flow chart of cryo-EM data processing of the Set2-xNCP^{WT} dataset (resolution: 3.3 Å). **b**, Representative micrograph of the cryo-EM dataset of the Set2-xNCP^{WT} complex. **c**, Representative 2D class averages of cryo-EM particles of the Set2-xNCP^{WT} complex. **d**, Cryo-EM density map (left panel) and atomic model (right panel) of the Set2-xNCP^{WT} complex. The cryo-EM map was segmented and colored according to the respective components of the Set2-xNCP^{WT} complex. Set2 could not be observed on the wild-type nucleosome, and the potential Set2-binding site was denoted with a dashed circle.

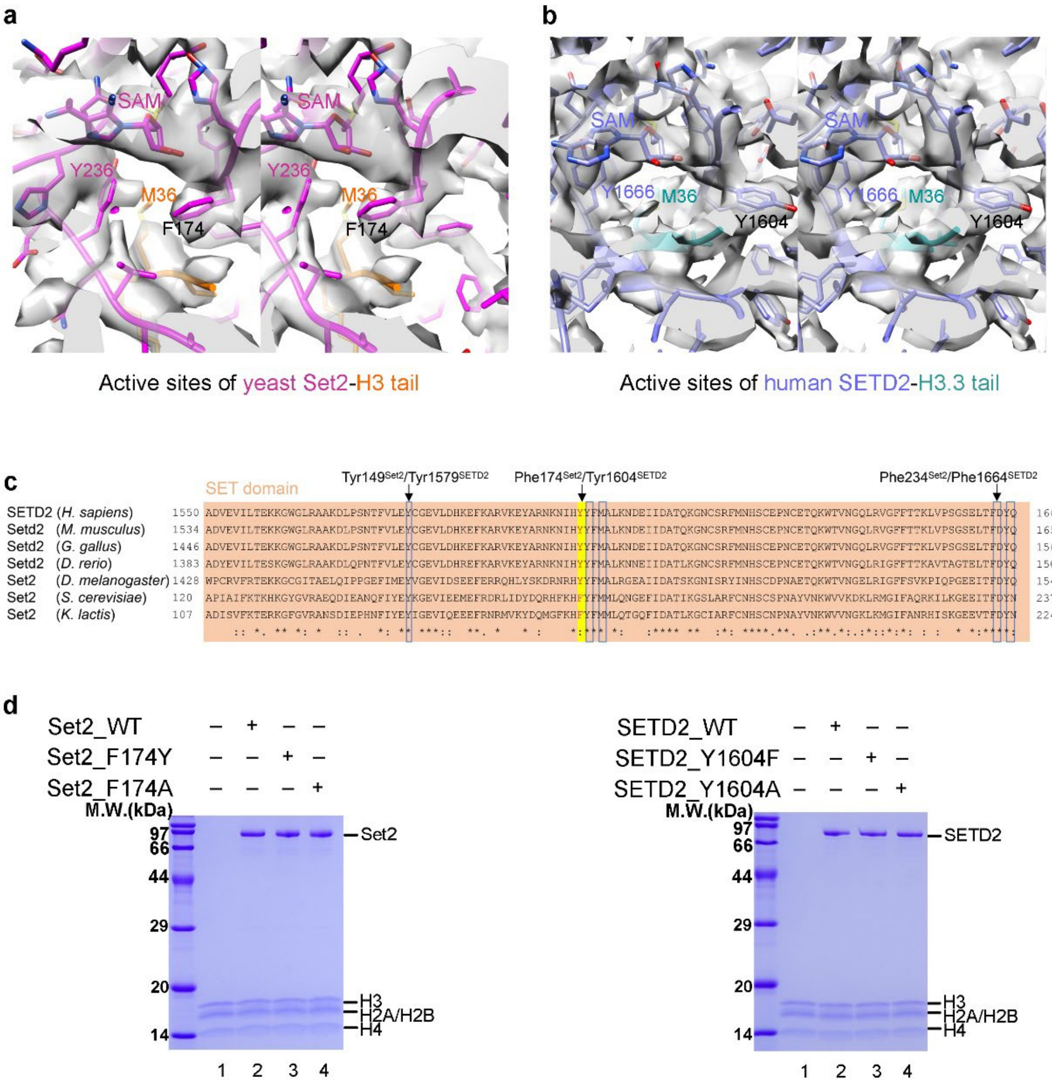

Supplementary Fig. S10. Structural and biochemical comparisons between human SETD2 and yeast Set2. a, The active site of Set2^{SET} within the Set2-xNCP^{H3K36M} complex, shown with the EM densities around the residue Phe174, the cofactor SAM, and the K36M mutation in stereo mode. **b**, The active site of SETD2^{SET} within the SETD2-hNCP$^{H3.3K36M}$ complex, shown with the EM densities around the residue Tyr1604, the cofactor SAM, and the K36M mutation in stereo mode. **c,** Sequence alignment of the SET domain of SETD2/Set2 from yeast to human, with the Phe174^{Set2}/Tyr1604^{SETD2} pair that is important to Set2/SETD2 activities highlighted in yellow. **d,** Input of the HMT reactions related to Fig. 4d.

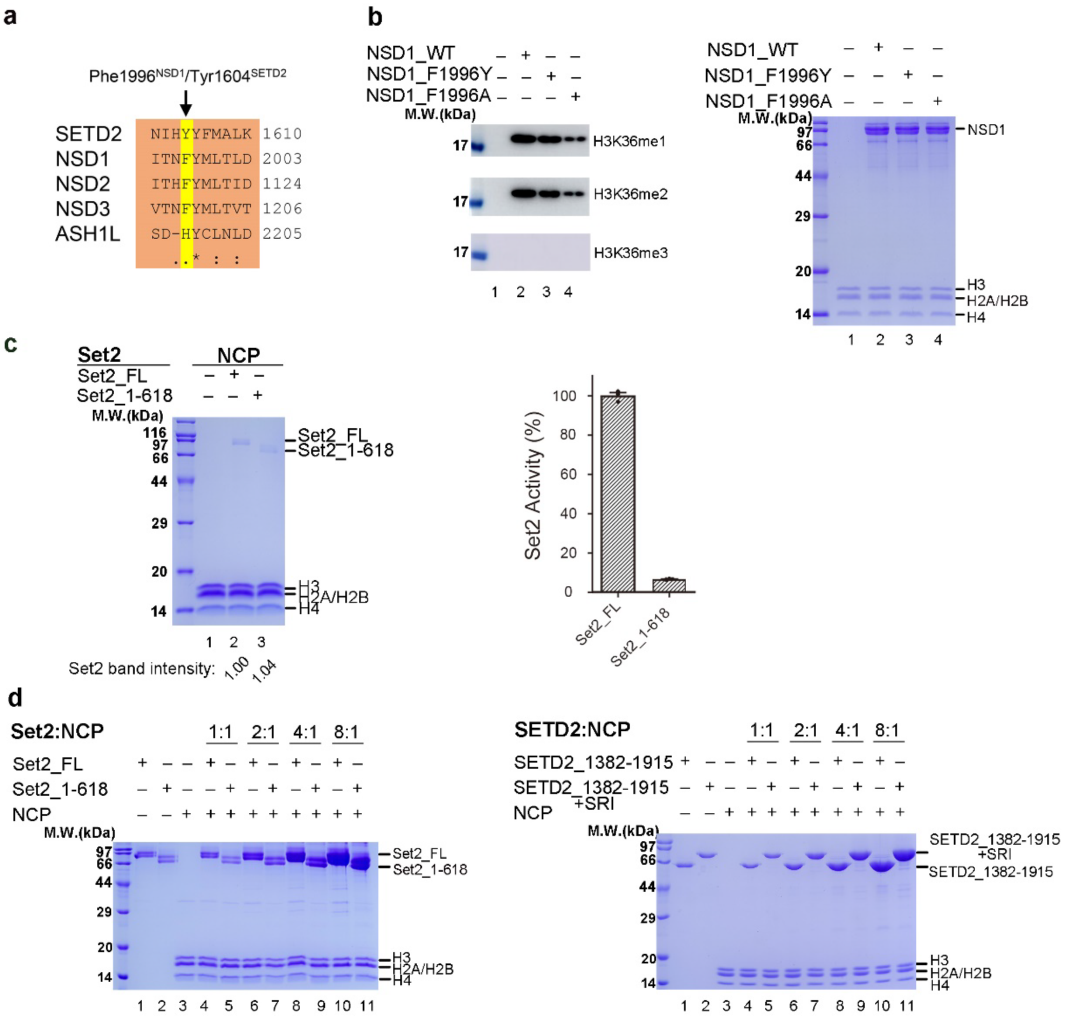

Supplementary Fig. S11. Biochemical comparisons between human SETD2, NSD1 and yeast Set2. a, Sequence alignment showing the counterpart of residue Tyr1604^{SETD2} in the other human histone H3K36 methyltransferases. **b,** HMT assays of wild-type and F1996Y/A-mutant NSD1 proteins against nucleosome substrates. Mono-, di-, and tri-methylation levels of histone H3K36 are determined with antibodies of H3K36me1, H3K36me2 and H3K36me3. Each assay was repeated at least three times with similar results. Right panel, input of the HMT reactions of NSD1 related to the left panel. The input amounts were quantified according to the band intensities of NSD1. **c,** Endpoint HMT assays performed with different truncations of yeast Set2. Left panel, input of the HMT reactions. The input amounts were quantified according to the band intensities of yeast Set2. Right panel, endpoint HMT assays of the different truncations of yeast Set2 against wild-type xNCPs. Error bars denote the standard deviation from the mean of three replicates. **d**, Input of the EMSA mixtures related to Fig. 4e.

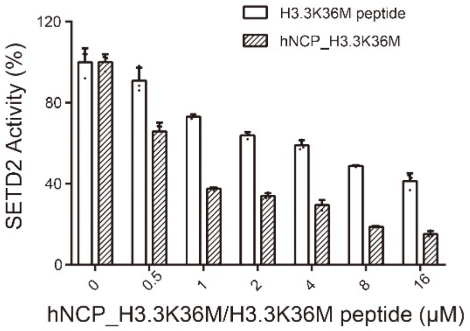

Supplementary Fig. S12. H3.3K36M mutation represses the deposition of H3K36me3 by SETD2 on nucleosome. HMT assays performed with SETD2 toward nucleosomes in the presence of increasing amounts of the H3.3K36M peptide or the H3.3K36M nucleosome. Error bars correspond to the standard deviation of three replicate assays.

Table S1. Cryo-EM Data Collection and Refinement Statistics

	**Human SETD2-hNCP$^{H3.3K36M}$ complex (EMD-31040) (PDB 7EA8)**	**Human SETD2-hNCP^{WT} complex (EMD-31041)**	**Yeast Set2-xNCP^{H3K36M} complex (EMD-31039) (PDB 7EA5)**	**Yeast Set2-xNCP^{WT} complex (EMD-31042)**
**Data collection and processing**				
Microscope	Titan Krios	Talos Arctica	Titan Krios	Titan Krios
Detector	K3	Falcon III	K3	K3
Voltage (kV)	300	200	300	300
Electron exposure (e-/Å^2)	50	40	50	50
Defocus range (μm)	-0.7 to -2.8	-1.0 to -2.0	-0.8 to -3.3	-0.8 to -2.6
Pixel size (Å)	1.10	1.24	1.09	1.10
Symmetry imposed	C1	C1	C1	C1
Initial particle images (no.)	4,166,530	335,223	2,913,095	653,517
Final particle images (no.)	154,984	99,922	225,474	177,138
Map resolution (Å)	3.1	3.7	3.3	3.3
FSC threshold: 0.143				
**Refinement**				
Initial model used (PDB code)	5JJY and 5X7X		6J99 and 5JJY	
Map sharpening B factor (Å^2)	-64		-70	
Model-to-map fit, map correlation coefficient	0.79		0.81	
Model composition				
Non-hydrogen atoms	12,861		13,764	
Protein residues	987		975	
DNA nucleotides	244		290	
Ligand	1		1	
R.m.s. deviations				
Bond lengths (Å)	0.011		0.008	
Bond angles (°)	1.034		0.85	
Validation				
MolProbity score	1.43		1.24	
Clashscore	2.28		2.28	
Poor rotamers (%)	0.0		0.0	
Ramachandran plot				
Favored (%)	93.2		96.2	
Allowed (%)	6.8		3.8	
Disallowed (%)	0.0		0.0	
